# Needs and challenges for assessing the environmental impacts of engineered nanomaterials (ENMs)

**DOI:** 10.3762/bjnano.8.101

**Published:** 2017-05-05

**Authors:** Michelle Romero-Franco, Hilary A Godwin, Muhammad Bilal, Yoram Cohen

**Affiliations:** 1 University of California Center for Environmental Implications of Nanotechnology, University of California, Los Angeles 6522 CNSI Building, 570 Westwood Plaza Box 957227 Los Angeles, CA 90095-7227, USA; 2 Department of Environmental Health Sciences, Fielding School of Public Health, University of California Los Angeles, Box 951772, 56-070 CHS Los Angeles, California 90095, USA; 3 California Nano Systems Institute, University of California Los Angeles, 6522 CNSI Building, 570 Westwood Plaza, Box 957227, Los Angeles, CA 90095-7227, USA; 4 UCLA Institute of the Environment and Sustainability, University of California, La Kretz Hall, Suite 300, Box 951496, Los Angeles, CA 90095-1496, USA; 5 Department of Chemical and Biomolecular Engineering, University of California Los Angeles, 5531 Boelter Hall, Los Angeles, CA 90095-1592, USA

**Keywords:** engineered nanomaterials, environmental impacts, risk assessment

## Abstract

The potential environmental impact of nanomaterials is a critical concern and the ability to assess these potential impacts is top priority for the progress of sustainable nanotechnology. Risk assessment tools are needed to enable decision makers to rapidly assess the potential risks that may be imposed by engineered nanomaterials (ENMs), particularly when confronted by the reality of limited hazard or exposure data. In this review, we examine a range of available risk assessment frameworks considering the contexts in which different stakeholders may need to assess the potential environmental impacts of ENMs. Assessment frameworks and tools that are suitable for the different decision analysis scenarios are then identified. In addition, we identify the gaps that currently exist between the needs of decision makers, for a range of decision scenarios, and the abilities of present frameworks and tools to meet those needs.

## Introduction

Engineered nanomaterials (ENMs) are increasingly being used in numerous industrial products and processes owing to their unique physicochemical properties. There are over 1000 nano-enabled consumer products [1], representing an estimated global and US markets of $1 trillion and $800 billion, respectively [2]. Applications of ENMS include, for example, nanomedicine (e.g., drug delivery, early diagnosis and therapy for chronic diseases) [3] and environmental remediation [4].

Given the rapid growth of the market for ENMs, there is concern about potential adverse impacts from possible exposures to ENMs during production, distribution, use, and disposal [5]. Human exposure to ENMs can occur through inhalation, ingestion or dermal absorption. Despite the fact that chronic health effects of ENMs have not been conclusively identified in human populations [6], animal studies suggest that the ENMs exhibit mechanisms of respiratory toxicity similar to those of ambient ultrafine particles (UFPs) [7]. Furthermore, in vitro and in silico studies [8–9] suggest that inhalation of some ENMs may cause additional adverse outcomes, such as damage to the respiratory tract, inflammation, and activation of signaling pathways. For additional routes of exposure, such as dermal absorption, existing evidence suggests that certain ENMs may penetrate the skin (e.g., cobalt nanoparticles in human volunteers and quantum dots ‘QDs’ in rat skin) and cause irritation (e.g., nano ZnO in zebrafish models) [10]. Oral exposure to ENMs can result in subsequent absorption in the GI tract and organ damage (e.g., nano Cu in mice via oral gavage damaged liver, spleen and kidneys, and nano ZnO caused necrosis of liver tissues and severe renal damage) [10]. Given the above concerns, decision-makers and relevant stakeholders are confronted with the need to identify and utilize reliable methods to ascertain environmental impacts related to the production, use and disposal of ENMs.

**Figure 1:**
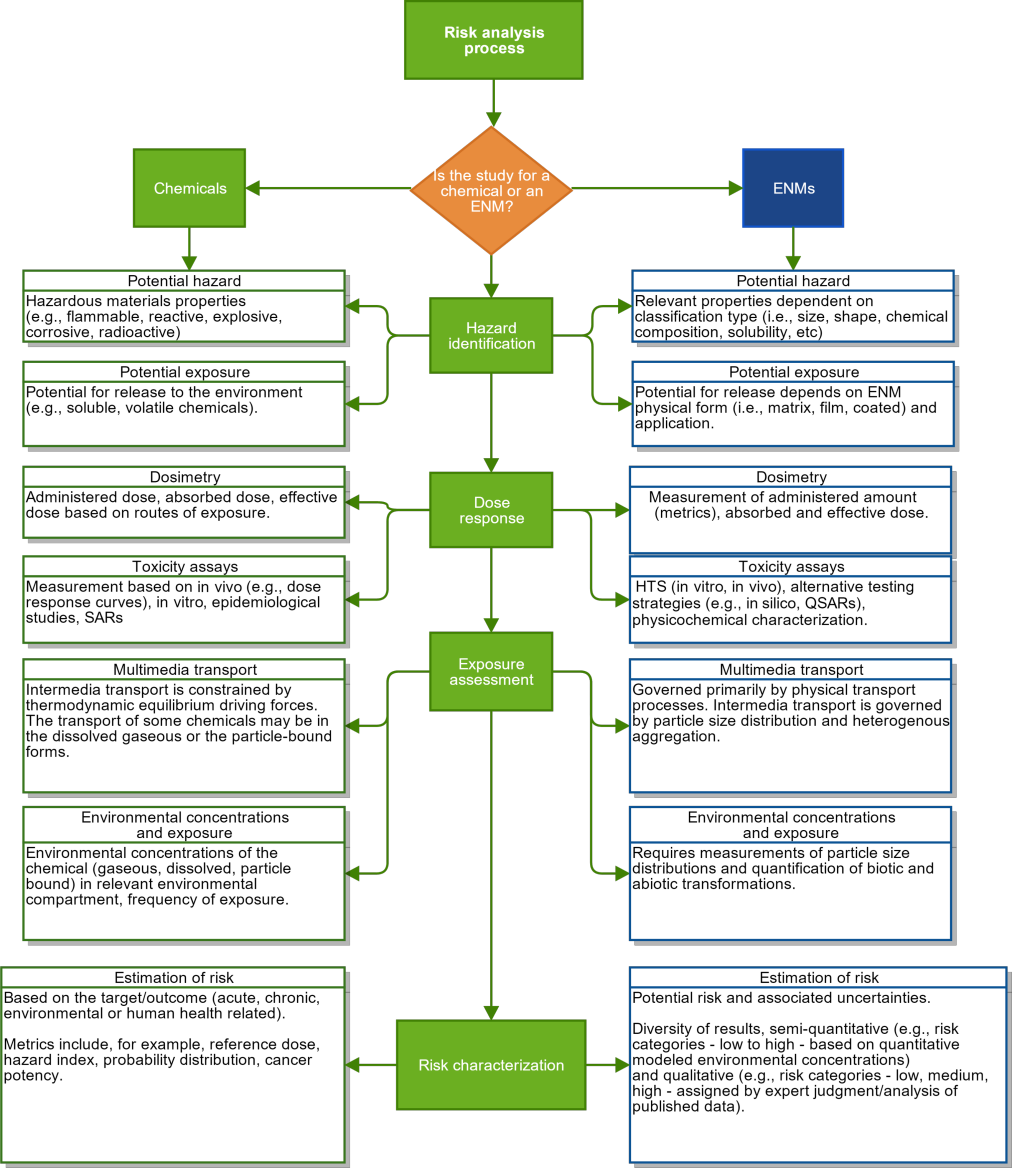
Challenges encountered at each step of the traditional risk assessment process for conventional chemicals and its relevance to ENMs.

The default process for evaluating the potential impacts of ENMs would be to use existing frameworks that were developed to assess the environmental health and safety (EHS) impacts of new chemicals and new industrial technologies more broadly. One such general framework is Environmental Impact Assessment (EIA), which was promoted within the National Environmental Policy Act (NEPA) in the early 1970’s [11] as a holistic approach that considers the environmental, social and economic implications of planned projects. Another existing general framework is “risk assessment” (RA) [12], which was developed to estimate human health related risks in a systematic manner based on toxicity, dose response curves and quantitative exposure assessment. These frameworks (i.e., EIA and RA) have also been incorporated into ecological risk assessment (ERA) [13], which is used to evaluate the likelihood of adverse environmental effects with focus on ecological receptors (e.g., biota, environmental compartments) [14]. However, application of these existing frameworks to ENMs is not straightforward. For example, although RA methods for chemicals are well established, their adoption and/or adaptation for ENMs would require consideration of various issues that include, but are not limited to the: behavior of ENMs in various media (e.g., dissolution, agglomeration/aggregation, adsorption); persistence (techniques to predict aspects of degradation of certain ENMs; transportation/distribution; predicted environmental concentrations (PECs) and transformation products and impurities; bioaccumulation; and effects/predicted no effect concentration (PNEC) [15–16] (see Figure 1). As a result, the implementation of RA for ENMs would be extremely costly and time consuming. Additionally, challenges such as the lack of information on background levels of naturally occurring nanoparticles and needed monitoring data on environmental concentrations of ENMs [17] restrict the application of traditional RA and EIA to ENMs. Furthermore, the adaptation of chemical RA to ENMs would require the development of data on: (i) ENMs hazard properties, (ii) ENMs dose-response and dosimetry metrics, (ii) production volume and emission rates (including modes of release) of ENMs, (iii) environmental transformations, and (iv) distribution of ENMs in the environment and associated multimedia exposure levels [15–16].

Due to data gaps, RA of ENMs that have been performed to date have frequently had to rely on expert judgment, which can result in ongoing debates about the validity of the results obtained from this approach [16,18]. Furthermore, the complexities of ENMs transformations (e.g., agglomeration, complex formation) make it difficult to quantify the relevant ENM absorbed and/or effective doses and complicate the development of dose–response relationships.

As defined by the United States Environmental Protection Agency (US EPA), “Risk characterization is an integral component of the risk assessment process for both ecological and health risks, i.e., it is the final, integrative step of risk assessment…risk characterization integrates information from the preceding components of the risk assessment and synthesizes an overall conclusion about risk that is complete, informative, and useful for decision makers” [19]. Evidence regarding the harmful impacts of chemicals has been traditionally addressed via toxicity and epidemiological studies, which allow risk assessors to establish causal relationships between exposure and effects. Risk characterization for chemicals entails quantitative integration of exposure assessment and dose–response information, and the metrics include the establishment of reference doses (e.g., to protect the susceptible population from harmful effects), hazard index and cancer potency factors. By contrast, the bulk of available toxicity data for ENMs are mostly based on in vitro assays and modeling approaches (e.g., quantitative structure activity relationships (QSARs)). For exposure assessment, the RA process for chemicals has been traditionally performed via laboratory studies, field monitoring, use of biomarkers, or fate and transport modeling. The RA process for ENMs, on the other hand, involves the additional complexity associated with having to address the impact of particle size distribution and agglomeration on the fate and transport and bio-uptake of ENMs by ecological receptors.

Given the complexity of quantifying environmental exposures to ENMs and the scarcity of toxicity data at the organism level, several alternative approaches have been proposed (as an alternative to RA or EIA) for assessing the potential environmental impacts of ENMs. Previous reviews of the above approaches [1,20–21] have provided insight into the various elements of the assessment methods, but did not assess whether they meet the needs for ENMs RA and the associated challenges faced by the multiplicity of stakeholders for specific decision-making scenarios. Here, we provide a critical assessment of

1. the types of decisions that different stakeholders in regulatory and non-regulatory environments need to make about the risk potential of ENMs and what types of tools they require;
2. which of the existing frameworks may be most suitable to address these needs; and
3. the gaps that exist between the needs of decision makers and the RA tools that are currently available.

## Methods

### Delineation of decision-making scenarios

To assess the relevance of the different risk assessment approaches, six different plausible decision-making scenarios were considered. The term “scenario” has been used across different fields and practices with different meanings and uses [22], ranging from management and planning (e.g., a description of future events to identify key variables and questions, trends and actors to identify strategic options) [23] to software design (e.g., envision of potential problems related to the use of the developed product) [24] and environmental assessment (e.g., assessment of pathways of events under a set of key assumptions (‘what if’?)) [25]. A common ground for the different uses of scenario analysis is in its application as a tool to study multi-disciplinary problems. Within the context of framework analysis in this review, “scenarios” are defined as a set of equally plausible contexts in a narrative form [22]. In the present review the example scenarios were selected with a focus on the United States landscape considering various frameworks reported in the published literature. The first four scenarios are related to manufacturing ENMs and occupational health and safety concerns, and the last two scenarios are related to registration of new ENMs and establishment of maximum allowable exposure levels. Evaluation of RA frameworks within the context of decision-making scenarios is particularly instructive in assessing the utility of specific RA methods [26]. The specific information needs for each decision-making scenario include:

1. definition of the intent of the analysis (e.g., selection of hazard identification, exposure assessment or risk characterization);
2. the level of resolution/type of the analysis result (e.g., qualitative categorization/prioritization of needed research or testing or quantitative information, for example, a permissible exposure limit (PEL));
3. the typical level of expertise of stakeholders who would be making the decision;
4. the type of data accessible to stakeholders (e.g., data reported from the literature, publicly available production reports, material safety data sheets (MSDS), etc.).

**Scenario I** reflects a process by which a company must decide whether to control exposure to workers during manufacturing or processing of ENMs. This is a common assessment carried out in industry to ensure occupational health and safety standards, require information about process details and potential exposures, and establish control practices.

**Scenario II** is for the establishment of safe exposure levels related to occupational health by a regulatory body (e.g., the US Occupational Safety and Health Administration (OSHA)). OSHA requires information to drive risk management (e.g., establishment and enforcement of occupational exposure limits (OELs) and permissible exposure limits (PELs) and guidance for compliance). The main needs of this scenario are to establish OELs/PELs for a specific class of ENMs for which the agency is required to provide evidence based exposure limits.

**Scenario III** is for a company manufacturing ENMs that has to decide whether risk associated with such ENMs or products containing ENMs is manageable and how to manage any potential risks. This decision-making scenario requires information/data regarding the potential for exposure throughout the ENM’s life cycle and the hazards it may pose to humans and the environment.

**Scenario IV** addresses the need for arriving at a decision by a company or regulatory agency for choosing the safest ENM out of a group of alternatives (ENMs or chemicals). In this scenario, the assessment of alternatives requires information regarding hazard posed to humans and the environment for all different alternatives as well as technical performance of the material for the intended application.

**Scenario V** focuses on decisions made by a regulatory body (i.e., the US EPA) about whether to control the use, release, or emissions of an ENM via a Significant New Use Rule (SNUR). This decision entails gathering substantial evidence that indicates any unreasonable risk to people or the environment given information about production volume, release, exposure potential and anticipated hazards. The SNUR must be justified considering:

1. the projected ENM volume of manufacturing and processing;
2. the extent to which ENM use changes the type or form of human and ecological receptors’ exposure to the ENM;
3. the extent to which the ENM use increases the exposure level and period;
4. the reasonably anticipated manner and methods of manufacturing, processing, distribution in commerce and disposal of a chemical substance [27].

**Scenario VI** focuses on decisions involving food, drugs or personal care products. In this scenario, a regulatory body (i.e., the US Food and Drug Administration (FDA)) needs to decide whether to allow registration of a new nano-enabled product in food, drugs and personal care products. In **Scenario VI**, examples are explored that pertain to cosmetics and new drugs containing ENMs [28]. While safety assessment is required for both product types, new drugs require a detailed Risk Evaluation and Mitigation Strategy (REMS) [29], including an estimation of population exposed to the drug, benefits from treatment with the drug, potential health risks, and if the drug represents a new molecular entity [30].

### Review of available frameworks in the context of specific decision-making scenarios

18 existing frameworks that are potentially useful for assessing the impacts of ENMs were evaluated. These frameworks can be categorized as follows: (1) hazard identification frameworks, (2) frameworks for environmental risk/impact characterization, and (3) frameworks for occupational risk characterization. The evaluated hazard identification frameworks were the Swiss Precautionary Matrix (SPM) [31], Risk Classification System based on Multi Criteria Decision Analysis (MCDA risk classification) [32–34], NanoRiskCat [35], the Decision-making framework for the grouping and testing of nanomaterials (DF4Nano grouping) [36], and the modified GreenScreen [37]. The evaluated frameworks for environmental risk/impact characterization were Life Cycle Analysis (LCA) [38–40], DuPont’s NanoRisk [41], US EPA’s Comprehensive Environmental Assessment (CEA) [42–43], NanoHAZ [44], Nanomaterial risk screening tool (NRST) [45], Engineered Nanoparticles – Review of Health and Environmental Safety: Human health and Ecological Risk Assessment (ENRHES RA) [46], Risk Quantification based on Probabilistic Mass Flow Modeling Analysis (PMFA Risk Assessment) [47], Forecasting of the Impacts of Nanomaterials in the Environment (FINE) based on Bayesian Networks (BN) [48], and Life Cycle Risk Analysis for nanomaterials (Nano LCRA) [49–50]. The assessed frameworks for occupational risk characterization were Risk based classification for occupational exposure control (Risk based OEL) [51], Risk Classification based on an Industry Insurance Protocol (RCIP) [52], CB Nanotool [53] and the Web-Based Tool for Risk Prioritization of Airborne Manufactured Nano Objects (Stoffenmanager Nano) [54].

The potential for using existing frameworks for environmental impact/risk assessment and other relevant health and safety assessment of ENMs was evaluated systematically (see Supporting Information File 1) by identifying the following characteristics for each framework:

1. The intent of the analysis based on the framework’s main elements of the risk assessment process (e.g., hazard identification, exposure assessment, and risk characterization);
2. The inputs required to conduct the analysis (e.g., environmental fate and transport data, physicochemical properties; toxicological information including dose–response information);
3. The outputs/results obtained from the analysis such as description of the outcome (e.g., predicted values of environmental concentration, probability of risk) and its category (e.g., quantitative value/magnitude, qualitative classification) relative to the analysis intent;
4. The intrinsic characteristics of the applied methodologies including, for example, the basis for the analysis (e.g., conceptual model, questionnaire, statistical model), settings or conditions for which the framework was designed (e.g., a specific geographical location, a particular working environment) and data used to support the design of the framework (e.g., experimental data, mechanistic studies, authors’ assumptions);
5. The capability of a framework to address data gaps (e.g., via consideration of expert judgment or modeling tools incorporated in the framework); and
6. The availability of software tools specifically designed to conduct the analysis.

Finally, a discussion is provided of the potential opportunities for improving and/or adapting current frameworks and to further develop recommendations for the development of future tools. Moreover, the reviewed frameworks and the corresponding required information were further evaluated within the context of the selected decision-making scenarios in order to identify remaining major challenges.

## Review

The basic characteristics of the 18 frameworks that were evaluated are summarized in Table 1. Below, we summarize the intent, inputs, outputs, intrinsic characteristics, ability to address data gaps, and availability of software tools for each of the frameworks. The frameworks were evaluated, as detailed in the following sections, according to their intended applications for hazard identification, characterization of environmental risk, and characterization of occupational risk.

**Table 1:** Summary of critical characteristics of existing risk assessment frameworks relevant to ENMs.

Name of the framework and developer	General description	Main output of analysis

Swiss precautionary matrix [31] (Swiss Federal Office of Public Health)	Decision tree/questionnaire about the ENM properties under consideration (e.g., dimensions), effects (e.g., reactivity, stability), and exposure/release potential (e.g., physical form of the ENM), suitable for pre-screening.	Classification of the hazard posed by the ENM into two main groups: A) no need for review of (unspecified) risk management measures; B) need for review of (unspecified) risk management measures or need for additional information.
Risk Classification System based on Multi Criteria Decision Analysis (MCDA risk classification) (various institutions) [32–34].	Systematic comparison of alternatives (ENMs) via outranking by assigning scores (e.g., qualitative scale of least-most desirable (1–4), subjective probability (0–100%), and quantitative measurement of size (0–100)) for pre-determined criteria related to hazard, including intrinsic ENM properties (e.g., agglomeration, reactivity/charge, critical function groups, contaminant dissociation and size) and factors affecting toxicity (bioavailability and bioaccumulation).	Categorical classification of the hazard (e.g.*,* toxic potential): very low, low, medium, high, and extremely high.
Hazard and exposure potential identification for ENMs in consumer products (NanoRiskCat) [35] (University of Denmark).	Decision tree/flowchart, where user answers “yes”, “no”, or “no data” to questions about the ENM of interest (e.g., physical form of the ENM applied to products, toxicity evidence, high aspect ratio, potential of transport across ecosystems).	Color-coded/categorical classification of the hazard posed by the ENM: the scale ranges from a grey color assigned to insufficient data, green-low hazard, yellow-medium and red-high.
DF4Nano grouping [36] (European Center for Ecotoxicology and Toxicology of Chemicals “ECETOC” (NGO)).	Theoretical framework presented in tables (e.g., threshold values obtained from published data and expert elicitation) to guide the user in the classification/prioritization of ENMs for additional testing/risk assessment.	Categorical classification of ENMs in four main categories: 1) soluble ENMs, 2) biopersistent high-aspect ratio (for which no additional testing is required), 3) passive ENMs, and 4) active ENMs (which require a further analysis/risk assessment).
Modified GreenScreen [37] (Clean Production Action Group (NGO)).	Hazard assessment framework designed to screen chemicals based on a range of toxicity endpoints and ENM physicochemical properties.	Categorical classification of ENMs in 5 main categories of aggregated benchmark (BM) scores to designate specific recommendations regarding ENM use based on the potential environmental and human health concerns as supported by available data.
Life Cycle Analysis (LCA) [38–40] (Various institutions).	Class of approaches that follow a product over its life stages, including: (a) material acquisition and purification, (b) manufacturing and fabrication, (b) commercial uses, and (d) end-of-life product management.	Environmental impacts of the product under analysis (e.g., effects on ecological receptors, potential CO_2_ emissions attributed to synthesis/manufacture of ENMs).
DUPONT’s Nanorisk [41] (Environmental Defense Fund (NGO) and DuPont (Industry)).	Systematic collection and organization of information, that can include a chemical process risk assessment (CPQRA) following AICHE guidelines in cases where sufficient quantitative data are available. CPQRA focuses on acute rather than chronic hazards. Risk in this system is defined as a function of a hypothetical scenario, the estimated consequence(s) of exposure, and the estimated exposure frequency.	Results for individual ENMs and scenarios are presented as lifecycle profiles that include information on physicochemical properties, ecotoxicity, and environmental fate to be used for risk management strategies. In cases where quantitative data are available, the results include a quantitative risk analysis of the industrial processes related to the ENM.
US EPA’s Comprehensive Environmental Assessment CEA [42–43] (US EPA).	Compilation of extensive information needed to inform a “collective judgment”. Experts must then analyze the information to provide guidance to decision makers such as research planners and risk managers. This framework is presented as a roadmap to guide the user in a systematic data collection and identification of critical data gaps.	Summary of available information regarding a specific ENM. Typically accompanied by an evaluation of the resulting information by a group of experts that provides recommendations for research priorities and risk management.
An Adaptive Screening-Level Life Cycle Risk-Assessment Framework for Nanotechnology (Nano LCRA) [50,55] (Vireo Advisors).	Systematic compilation of information (e.g., properties, potential exposure and hazard of ENMs through all life cycle stages for a particular product) guided by a “roadmap” that is further analyzed by experts.	Summary of information with main findings/expert judgment based on those findings and indication of further information needs.
Ranking initial environmental and human health risk: Nano HAZ framework [44] (University College Dublin).	Process for developing qualitative risk rankings, including ecological risk and/or human health risk, for ENMs. Risk rankings reflect Bench Mark Dose (BMD) calculations, which are based on published/available data.	Categorical classification of ENMs into relative risk ranking groups: 0–2 (low environmental or health risk on a relative basis), 3–4 (concentrations that require monitoring and potential action), 5 + (environmental concentration above those provisional regulatory and toxicological limits as set in this study).
Nanomaterial risk screening [45] (University of British Columbia and Decision Research (non-profit organization)).	The framework guides the user through the process of assigning risk groups to ENMs. The categories are determined based on comparisons between data for the ENM under analysis to a reference set of information (tables) provided by the framework.	Categorical classification of ENMs in risk groups, where lowest concern = 1 and highest concern = 5.
Engineered Nanoparticles – Review of Health and Environmental Safety: Human health and Ecological Risk Assessment (ENRHES RA) [46] (European Commission Institute for Health and Consumer Protection).	Risk assessment of specific ENMs based on 90-day exposure studies and likely environmental concentrations determined by probabilistic models.	Ratio of the predicted environmental concentration for ENM of interested to the (predicted) concentration at which there is no effect (related to human health; PEC/INEC).
A risk quantification based on probabilistic mass flow analysis (PMFA risk quantification) [47] (Swiss Federal Laboratories for Materials Science and Technology (EMPA)).	Risk assessment for ENM of interest that combines predicted environmental concentrations (determined via probabilistic modeling) with a species sensitivity distribution (e.g., probability distribution of harmful effects shown at different concentrations for a given ENM).	Quantitative measure of risk calculated from the product of the probability of critical environmental concentrations and the probability that organisms would potentially be negatively impacted by such concentrations.
Bayesian Networks based FINE (Forecasting the Impacts of Nanomaterials in the Environment applied to nanoAg) [48] (Center for the Environmental Implications of NanoTechnology (CEINT) at Duke University).	Method for calculating the probability of risk for an ENM of interest using a Bayesian Network designed with inputs from expert judgment.	Modified version of a deterministic risk quotient (quantitative measure of risk) in a probabilistic expression.
Risk based classification for occupational exposure control (Risk based OEL) [51] (Nanotechnology Research Center (NTRC) and National Institute for Occupational Safety and Health (NIOSH)).	Process for quantitatively assessing the risk associated with the ENM of interest by applying benchmark doses (BMD).	Percent excess risk related to a specific health outcome as a result of exposure to the ENM under analysis.
Risk classification based on an Industry Insurance Protocol (RCIP) [52] (Rice University, Golder Associates Inc and XL insurance).	Comparison of scores assigned to characteristics of the target industrial process with pre-established scores from an insurance protocol.	Relative risk ranking for the ENM process compared to conventional industrial chemical process.
Control Banding: CB Nanotool [53] (Delft University of Technology).	Classification based on characteristics of the potential for exposure during preparation the ENM of interest (e.g., estimated amount of ENMs, dustiness/mistiness, number of employees with similar exposure, frequency and duration of operation) and properties related to hazard of the ENM (e.g., surface chemistry, particle shape and diameter, solubility, carcinogenicity, reproductive toxicity, mutagenicity, dermal hazard potential).	Risk banding of occupational risks. The risk bands are intended for developing recommendations risk management strategies for exposure control (e.g., RL 1: general ventilation; RL 2: fume hoods or local exhaust ventilation; RL 3: containment; RL 4: seek advice of environmental health specialist).
Web-Based Tool for Risk Prioritization of Airborne Manufactured Nano Objects (Stoffenmanager Nano) [54].	Classification based on the characteristics of the potential for exposure during preparation of the ENM of interest (ENM size, aspect ratio, handling, background exposure, duration, frequency) and properties related to hazard (e.g., toxicity) associated with the ENM.	Priority banding where the bands indicate the priorities for risk management.

### Hazard identification frameworks

The following four frameworks for identifying hazards associated with engineered nanomaterials were assessed: (a) the Swiss Precautionary Matrix, (b) “Risk” Classification Systems Based on Multi Criteria Decision Analysis, (c) NanoRiskCat, and (d) Decision-making framework for the grouping and testing of nanomaterials. Although several of the above approaches include “risk” in their titles, in practice they have been used either to solely assess hazard and/or do not yield a combined risk score. As a result, the above frameworks that are summarized below are considered in the present review as a category separate from those frameworks that have been used to identify risks associated with ENMs.

**The Swiss Precautionary Matrix** (SPM) was designed as a response to the Swiss Action Plan on Synthetic Nanomaterials (SAPSN) to employ existing information to identify potential harmful impacts of synthetic nanoparticles on health and the environment [31]. The SPM, which is available as a web-tool [56], is not designed to be a comprehensive risk assessment framework, but rather to provide an initial screening approach to determine the required measures for safe handling of nanomaterials in Switzerland [31]. Prior to evaluating a specific ENM or a nano-enabled product using the SPM, the analyst has to assess whether that material or product meets the definition of “nano-relevant” using the European Union regulatory recommendation of 2011 [57]. According to this recommendation, a nanomaterial is defined as “an unknown material containing primary particles in an unbound state or as an aggregate or as an agglomerate and where, for 50% or more of the primary particles in the number size distribution, one or more external dimensions is in the size range 1–100 nm or if the number size distribution is unknown”. A material is also considered to be “nano-relevant” by this definition if its specific surface area per unit volume is greater than 60 m^2^/cm^3^, or it is a material that consists of fullerenes, graphene flakes or single wall carbon nanotubes.

For materials considered to be nano-relevant, the SPM tool can be used to develop scores representing levels of concern for the following parameters: potential effect (W), potential exposure of humans or environmental release (E), and available information on the material’s life cycle (I). Threshold values and limits for each of these parameters (W, E, I), as determined from the peer-reviewed literature are specified in the SPM guidance document, which is provided online by the tool developers [31]. The potential effect (W) is a score assigned on the basis of the ENMs’ reactivity (e.g., redox activity, catalytic activity, oxygen radical formation potential or induction potential for inflammation reactions) and stability (e.g., half-life of the nanomaterial in the human body, or under environmental conditions). For example, a metal oxide nanoparticle with a conduction band energy that is much lower relative to the redox potential of biological molecules would be given the lowest score (1) on a scale of 1–9 (where 1 is low, 5 is medium and 9 is high). Likewise, a score is assigned for the ENM period of stability (e.g., 1, 5 and 9 for hours, days-weeks, and months respectively). The potential for human exposure to a given ENM and its environmental release (E) is assessed based on: (a) the carrier material of the ENM and availability of the ENM for release, (b) the maximum possible extent of human exposure via inhalation based on the daily amount of nanomaterial with which a worker comes into contact; (c) the maximum ENM input into the environment during manufacture via wastewater, exhaust gases or solid waste; and (d) the available information on the ENM life cycle. Finally, the score for (d) the available information on life cycle (I) is assigned based on the answers the user provides to the following questions: (i) Is the ENM’s origin (i.e., starting material(s)) is known? (ii) Is sufficient information available to complete the SPM based on the ENM starting materials? (iii) Are users of the ENM known? and (iv) Is the composition/purity of the ENM known or can be estimated?

Once the categories for each of the assessed parameters has been determined, an overall score for the nano-relevant material is calculated, which is expressed as a Precautionary need = *f* (N, W, E, I) [31]. The resulting score represents a measure of the need to review existing measures or evaluate new measures of risk management related to the nanomaterial. It should be noted that the SPM framework allows for updating data/information given that calculated scores can be high when there is lack of knowledge. Overall, SPM is a useful approach for setting priorities for action related to nano-relevant materials. However, the SPM tool does not identify specific control measures for risk reduction nor quantifies the risk/impact posed by the nano-relevant materials in question.

**“Risk” Classification Systems Based on Multi Criteria Decision Analysis (MCDA)** has been used as an analytical framework for environmental hazard assessment and/or management for ENMs, which can also aid in decision support or decision analysis [58]. Although this approach has been termed “Risk Classification by MCDA”, to date, use of this approach has primarily focused on hazard identification; hence for the purpose of this review it is categorized as a “hazard identification” approach [32,58]. The MCDA process involves [32] identification of stakeholders and evaluation criteria, elicitation of MCDA parameters (e.g., establishment of weights and thresholds), model execution using available software tools [59], and interpretation of results. It is noted that MCDA is often limited with regard to addressing data gaps, since scores must be provided by the assessor or via expert elicitation. Integration of MCDA with Life Cycle Analysis (LCA) and RA has been proposed to compare the impact of ENMs across life cycle stages [60]. However, case studies based on this integrative approach have not yet been reported (as of September 2016).

An interesting MCDA example of classifying the risk potential of ENMs is the “stochastic multi-criteria acceptability analysis” (SMAA-TRI) approach [34]. In the above approach, ENMs are classified into “risk” categories (e.g., very low, low, medium, high and extremely high risk) to provide recommendations for additional testing prior to ENM use in consumer products [34]. In the SMAA-TRI method, the highest scoring category (i.e., extremely high risk) is assigned to ENMs that have high scores for the majority of the criteria described below. The SMAA-TRI was utilized [61] to compare a set of ENMs (called “alternatives” in the framework) based on selected hazard and toxicity criteria (e.g., extrinsic ENM properties such as agglomeration, reactivity/charge, critical functional groups, contaminant dissociation and size; evidence of toxicity; and other factors related to toxicity such as bioavailability and bioaccumulation). This outranking method has the advantage, when criteria metrics are not easily aggregated, of providing qualitative metrics for ENM ranking (e.g., “most-least” favorable) [32]. The SMAA-TRI approach has been demonstrated for ranking of C60, multi-walled carbon nanotubes (MWCNTs), CdSe, Ag nanoparticles (NP), and Al NP according to the following scales: size (quantitative scale 1–100 based on literature review for the studied ENMs); agglomeration, reactivity/charge, critical functional groups and contaminant dissociation (qualitative scale of 1–5, where scores are assigned based on expert judgement with 1 representing the most favorable score as judged based on the perceived hazard/toxicity (i.e., lower score for less harmful/toxic ENM) and 5 the least favorable/more toxic); and toxicity evidence, bioavailability, and bioaccumulation (scale of 0–100 of a “subjective” probability scale constructed by the authors based on their expert judgment). In the proposed approach, the authors followed the scoring with a Monte-Carlo simulation to sample from a given probability distribution for each parameter to arrive at probabilities for the ranked ENMs for each of the categories. Such analysis suggested that CdSe was of greatest concern among the analyzed ENMs, ranking in the high-risk category with a 98% probability. In contrast, the ranking for C60 was fairly evenly divided between medium risk (51% probability) and high risk (49% probability), and Al NP was fairly evenly divided between medium risk (34% probability) and low risk (33% probability) [34]. As illustrated by the above case study, the Risk Classification MCDA framework is useful in a context where the ENMs hazards are known and can be reasonably or rationally grouped within categories for the intended ranking. However, the assignment of ENMs’ properties or hazard traits involves subjective expert analysis and therefore may be biased depending on the knowledgebase available to the assessor.

**NanoRiskCat** is a roadmap/flowchart designed as a first-tier approach to assess and communicate the hazard and exposure potential of ENMs that are used in consumer products [35]. In the above approach hazard and exposure potentials are assessed individually and are not combined to yield a risk score. Thus, in the current review, NanoRiskCat is categorized as a “hazard identification framework”. This framework typically requires expert judgement in order to interpret the available data. In addition, use of this framework by individuals other than the developers is currently limited given the present unavailability of a software implementation of the framework. Nonetheless, this framework could serve to aid companies and regulators for assessing the potential exposure, human health and environmental hazards associated with specific ENMs.

The NanoRiskCat framework leads the assessor through a series of questions that guide through the process of qualitatively classifying the hazard and exposures potential of the ENM of concern. Qualitative Classification is expressed in terms of a color code where red, yellow, green and gray indicate high, medium, and low potential hazard/exposure, respectively, while gray signifies that data are insufficient for an assessment. Questions are then posed to allow one to classify the hazard and exposure potentials; such questions also include queries regarding the physical form of the ENM and potential receptors (e.g., professional-end users, consumers and/or environment) that could be exposed to the ENM. The framework includes questions about the potential hazards of the ENM with respect to human health (e.g., evidence of acute toxicity, germ cell mutagenicity, carcinogenicity, reproductive toxicity) and environmental hazards (e.g., adverse outcomes to aquatic and terrestrial species). Based on answers to the posed questions, NanoRiskCat classifies ENMs into three categories of potential exposure (high, medium and low) [62–63]. The potential for ENM human hazard is also evaluated based on answers to questions about the ENM aspect ratio (e.g., a high aspect ratio ENM is categorized immediately as high), evidence of adverse outcomes related to acute and chronic effect posed by the ENM (e.g., evidence to support genotoxicity, neurotoxicity, carcinogenicity, and/or cardiovascular, respiratory toxicity). Environmental impacts are also assessed based on bioaccumulation, persistence, as well as dispersibility and other “warning signs” of potential hazard [64]. Given the above, it can be stated that qualitative results obtained via NanoRiskCat are intended to be a tool for risk communication strategies.

The use of NanoRiskCat was demonstrated for categorization of the following ENM containing products: cleansing soap (containing nano Ag), tennis rackets (CNTs), automotive oil (Fullerene C60), and sunblock (nano ZnO), among others [35]. NanoRiskCat analysis concluded that sunblock and cleansing soap were in the category of overall red/high exposure potential for human and for environmental hazards [35]. The tennis racket, as a source of ENMs, on the other hand, was categorized as being of low potential exposure. However, since the tennis racket contained CNTs it was designated in the medium/high category for human and environmental hazard. As demonstrated in the case study [35], the NanoRiskCat framework can be a useful tool to qualitatively identify areas of concern (e.g., ecological and/or human health hazards) through the analysis of published information. However, the approach is not built for direct analysis of quantitative data or handling of areas of missing information.

The **Decision-making framework for the grouping and testing of nanomaterials (DF4Nano grouping)** was designed by the European Center for Ecotoxicology and Toxicology of Chemicals (ECETOC) “Nano Task Force” as a regulatory framework to guide the users on grouping ENMs to make human health hazard assessment and identify information needs/research priorities for inhaled ENMs [36]. This framework leverages the concept of “read-across”, which allows data gaps to be filled assuming that ENMs with similar structures and/or physicochemical properties will exhibit similar hazard profiles [36]. The Nano Task Force proposed that this framework could be useful for categorizing substances into common groups based on similarity of structural and physicochemical properties that induce similar patterns of toxicity.

In DF4Nano, ENMs are grouped into four main categories: soluble ENMs, biopersistent high aspect ratio (HAR) ENMs, passive ENMs, and active ENMs. Soluble ENMs are defined as ENMs with a water solubility that exceeds 100 mg/L or not water-soluble but soluble in biological media and/or if the ENM has a pulmonary half-life of less than 40 days. For soluble ENMs, no further nano-specific sub-grouping is specified and read-across of the properties of the dissolved materials to the corresponding bulk materials is applied. Biopersistent high aspect ratio (HAR) ENMs are defined as ENMs with an aspect ratio less than 3:1, a length greater than 5 μm, a diameter less than 3 μm, and an aqueous dissolution rate (suggesting biopersistence) greater than 100 mg/L or a pulmonary half-life upon intratracheal instillation greater than or equal to 40 days. Passive ENMs are those materials considered to be of very low or no hazard potential by virtue of containing less than 0.1% toxic components, low surface reactivity (e.g., based on ferric reducing ability of serum or cytochrome C), high dispersibility (based on an average aggregation number (AAN) ≥ 3), no cellular effects observed at a surface area ≤10 µg/cm^2^, and low toxic potency (i.e., a no adverse effect concentration (NOAEC) in short-term inhalation studies (STIS) >10 mg/m^3^). Active ENMs are those that either do not meet the criteria for soluble ENMs, biopersistent high aspect ratio ENMs, or passive ENMs, or that meet the criteria for multiple categories, assuming that the NOAEC for the ENM in STIS is ≥610 mg/m^3^. For ENMs of group 4, further sub-grouping is required according to the degree of mobility in air (dustiness) and in physiological fluids (dispersibility), as well as on the uptake, biopersistence, and biodistribution as determined in vitro and in vivo short term inhalation studies (STIS).

The DF4Nano Grouping is based on data provided by the analyst for the ENMs of interest through “tiers” or information filters, where specific thresholds are set for intrinsic material properties (e.g., water solubility, primary particle size, surface area, composition, crystallinity, and surface chemistry); system-dependent properties (e.g., dissolution rate in biological simulation fluid (BSF), release of toxic ions, size in relevant media and dispersibility); biopersistence (e.g., property of the ENM to persist in a cell, tissue, organ or organism as a proxy of pulmonary retention); uptake and biodistribution (e.g., evidence of alveolar uptake and subsequent distribution through the pulmonary system); and cellular (e.g., membrane damage including cationic phagolysosome damage, generation of reactive oxygen species (ROS), oxidative stress, redox activities, etc.) and apical toxic effects (e.g., respiratory effects shown in short-term inhalation studies).

The initial tier (0) focuses on gathering data regarding intrinsic material properties (e.g., water solubility, primary particle size (PPS), surface area, composition, crystallinity, and surface chemistry). In tier 1, the ENM can be assigned into one of the following groups of intrinsic material properties: water solubility, particle morphology (PPS and shape, including aspect ratio and surface area) and chemical composition. Tier 2 focuses on the ENM’s i) intrinsic properties and those linked to the ENMs functionality in the environment, (e.g., surface reactivity, dissolution rate, and dispersibility), ii) intended use, release and exposure, iii) uptake, biodistribution and biopersistence, and iv) biophysical interactions and cellular effects [36] to assign non-soluble ENMs to one of the following groups: biopersistent high aspect ratio (HAR) ENMs, passive ENMs, or active ENMs. Analysis within tier 2 is meant to indicate whether the ENM should be classified as either a biopersistent HAR ENM or an active ENM. Tier 3 is reached if the ENM has not been classified within any of the groups of tiers (1) and (2) or to confirm/revise the assignment of ENMs to the resulting category. Tier 3 includes a confirmation of in vivo toxic effects, which are considered higher in ranking than in vitro effects, to define and refine additional information needs. The specific toxicological information assessed in tier 3 includes: lung burden, systemic uptake, in vivo biopersistence, biodistribution, apical toxic effects and toxic potency, as assessed by STIS, in addition to ex vivo genotoxicity screening.

The application of the DF4Nano Grouping has been proposed [36] as a resource where physicochemical characterization and toxicity data are available for the ENM under consideration, or for those ENMs with similar properties to those for which toxicological information is available. In cases involving novel ENMs or where physicochemical characterization data are lacking, the application of DF4Nano requires additional ENM characterization. Also, given that exposure assessment is not performed in this framework the applicability of DF4Nano is suitable where qualitative assessments may suffice.

A **modified GreenScreen** tool [65] was recently developed [37] following the original GreenScreen approach advanced by the Clean Production Action Group to assist in conducting chemical hazard assessment. The approach incorporates aspects of the US EPA’s Design for the Environment (DfE) Alternatives Assessment Criteria for Hazard Evaluation and the Globally Harmonized System (GHS) of Classification and Labelling of Chemicals [37,65]. GreenScreen was modified for application to ENMs by including collection of physicochemical properties of the target nanomaterials (e.g., agglomeration and or aggregation, chemical composition, purity, shape, surface area, surface chemistry (including composition and reactivity)) [37]. The various studies from which information on the nanomaterials (properties and toxicity endpoints) is compiled are then assessed with respect to the reliability of the provided information. Briefly, the application of the modified GreenScreen approach, which is available as an online software tool, entails the following steps:

1. collection of publicly available data for 18 parameters that are relevant to hazard outcome (both chronic and acute) associated with the hazard endpoints of the target chemical,
2. expert evaluation of the collected data to assign “Benchmark Scores” (e.g., low, medium or high concern) or “DG” for data gaps to each of those 18 hazard endpoints;
3. assigning an aggregated benchmark (BM) score to categorize recommendations with respect to the material use.

The proposed five categories are as follows: BM1 is for a substance of very high concern as defined by U.S., Canadian and European regulatory bodies, BM2 and BM3 designate a material that can be continued to be used but safer substitutes are desirables as the nanomaterial may present human health concerns, BM4 is for a material that represent low hazards to humans and the environment, and a fifth category (BM-U) where information is insufficient to assign a score.

In a case study developed by Sass et al. [37], two types of nano-sized Silver, AGS-20 and low soluble nano-Silver, were compared to non-nano Silver (conventional Silver). Analysis using the modified GreenScreen tool suggested that low soluble nano-Silver and conventional Silver were of category BM-1 given evidence of high persistence and high ecotoxicity. In contrast, the lack of data for AGS-20 suggested classification of BM-U. As the above study notes [37], the modified GreenScreen tool is not intended for quantitative risk assessment, but rather as a suitable means for rapid screening to identify data needs and to compare available hazard information for ENMs.

### Frameworks for characterization of environmental risk

In evaluating frameworks that were designed to explicitly assess both hazard and exposure potential and to yield a net measure of risk potential, the present review focused on first considering frameworks that were designed to characterize environmental risk (including risks to humans due to environmental multimedia exposures) and then those designed to characterize occupational risk. Nine different frameworks for characterization of environmental risk of ENMs were assessed: (a) Life Cycle Analysis, (b) DuPont’s Nano Risk Framework, (c) the US EPA’s Comprehensive Environmental Assessment (CEA) Framework, (d) NanoHAZ, (e) Nanomaterial risk screening tool (NRST), (f) the Engineered Nanoparticles – Review of Health and Environmental Safety: Human Health and Ecological Risk Assessment (ENRHES RA), (g) Risk Quantification based on Probabilistic Mass Flow Analysis (PMFA risk quantification), (h) Forecasting of the Impacts of Nanomaterials in the Environment (FINE) based on Bayesian Networks (BN), and (i) Life Cycle Risk Analysis for Nanomaterials (Nano LCRA).

(a) **Life Cycle Analysis (LCA)** refers to a class of approaches that follow a product over its life stages, including: (a) material acquisition and purification, (b) manufacturing and fabrication, (b) commercial uses, and (d) end-of-life product management [66]. LCA is rooted in assessing environmental impacts. Examples of impacts that have been assessed previously using LCA include climate change, smog creation, eutrophication, toxicological stress on human health and ecosystems, depletion of resources that occur as a consequence of releases into the environment, and consumption of resources [67]. According to the Society of Environmental Toxicology and Chemistry (SETAC), LCA consists of the following steps: i) goal scope and definition (e.g., establishment of the product under analysis and study objectives); ii) life cycle inventory analysis (e.g., tabulation of emissions and consumption of resources at each life stage of the product); iii) life cycle impact assessment (e.g., assessment of the impacts at each life stage of the product, which depend on the scope of the LCA); and iv) life cycle improvement assessment (e.g., a review of the LCA results to reduce impacts related to the product under analysis) [67].

The applicability of LCA to assess the environmental impacts of ENMs has been the subject of different reviews [39–40,68], and the integration of LCA with risk assessment has also been suggested as a tool that could inform the development of nano-enabled products that are “safer by design” [5]. For example, Grieger et al. [68] qualitatively analyzed published case studies of ENMs RA and LCA. Their analysis demonstrated the differences between these two approaches: LCA provides an assessment of environmental impacts of a product/system while RA provides an assessment a particular substance or component of a complex material. Hischier et al. [40] reviewed LCA case studies of several ENMs (e.g., CNTs, single walled CNTs, fullerenes, quantum dots and TiO_2_) and nano-enabled products (e.g., dye containing nano-TiO_2_ and carbon powder, t-shirt with nano Ag coating, and polymer composite) to assess the potential contributions of material production to CO_2_ emissions. Most of the reviewed studies focused primarily on inventory of CO_2_ emissions or energy analysis [40]. An exception was a partial LCA and aquatic ecotoxicity impact assessment of carbon nanotubes (CNTs) reported by Eckelman et al. [38]. This latter study compared the environmental impacts (in freshwater) of chemical releases resulting from the manufacture (e.g., arc ablation, chemical vapor deposition (CVD), and high-pressure carbon monoxide (HiPco)) for a hypothetical scenario in which CNTs and chemical releases are associated with the production of CNTs. The environmental impact of CNTs was quantified via a characterization factor (i.e., *CF* = effect factor × fate factor × exposure factor) calculated as per the methodology of the fate and transport module of the USEtox model [69]. The aquatic environmental impact of the release of chemicals released to freshwater due to CNT manufacturing was assessed based on previously reported data [70] and LCA software (Sima Pro 7.3) [38]. In the above approach, the effect factor was defined as the ratio of the potentially affected fraction (PAF) of aquatic organisms and average EC_50_’s for the evaluated aquatic species. The fate was quantified as the residence time (days) of the CNTs or related chemicals in freshwater expressed as per the USEtox model. Here it is emphasized that USEtox was not developed to specifically describe the fate and transport of particles or particle-bound chemicals; thus, its extended application to ENMs was based on heuristic assumptions and approximations. In the presented case studies, two different hypothetical release scenarios were considered for two hypothetical cases in which either 100% (“worst case”) or 2% of the total produced CNTs were assumed to be released to freshwater. It was concluded that under the “worst case” scenario the expected environmental impacts of CNTs would be equivalent to that which would result from chemicals released to the environment during the manufacture of CNTs. However, under the 2% release scenario, the expected environmental impacts of CNTs were assessed to be several orders of magnitude lower than for chemicals released during the manufacture of CNTs. Hence, further research was recommended for the purpose of developing safer manufacturing processes for CNTs [38]. It is important to note that LCA offers a myriad of options for analyses of ENMs that have to be considered on a case-by-case basis. Depending on the scope of the assessment when sufficient data are available regarding ENM properties, fate and transport parameters, emissions, and toxicity/hazard then LCA could be performed to a reasonably approximate level via the USEtox model. The results of such analysis must be evaluated cautiously given that USEtox is a model designed for dealing with organic chemicals and does not consider the complex environmental fate and transport behavior and toxicity of ENMs.

(b) **DuPont’s NanoRisk Framework**, developed based on a joint effort by the Environmental Defense Fund and DuPont [41], is a guide to presenting questions and request for information that should be considered by an organization to evaluate the risks associated with specific ENM applications. NanoRisk is a qualitative framework that guides the development of informational profiles (e.g., properties, hazards and exposures associated with a nanomaterial and its application) for the target ENMs throughout their lifecycle. The output is a worksheet that includes information on:

1. material description and application (e.g., technical name, commercial name, common form),
2. ENM Profile Lifecycle(s) which consists of ENM Lifecycle Properties (ELP), ENM Lifecycle Hazard, and ENM Lifecycle Exposure (ELE) Profiles.

The NanoRisk ELP Profile includes ENM physicochemical properties such as chemical composition, surface coating, molecular structure, crystal structure, physical, form/shape, particle size, size distribution and surface area, agglomeration state, particle density, ENM bulk density, porosity, dispersibility, solubility in water and biologically relevant fluids, surface charge, and surface reactivity. The ELH profile includes acute hazard/toxicity information for the target ENM, and the ELE profile focuses on workers’ exposure to ENM during the industrial process.

NanoRisk is useful in guiding the analyst in gathering information needed to assess the potential risk associated with the ENM of interest following the Chemical Process Quantitative Risk Assessment (CPQRA) approach [71]. CPQRA is a methodology applied in the chemical, petrochemical and oil processing industries to evaluate the overall process safety rather than a specific chemical substance or ENM [71]. CPQRA consists of six steps:

1. definition of potential incidents (e.g., qualitative hazard analysis),
2. evaluation of potential consequences of the incidents (e.g., via vapor dispersion modeling and fire and explosion effect modeling),
3. estimation of the potential incident frequencies (e.g., via databases),
4. estimation of the incident impacts on people, environment and property,
5. estimation of the risk (e.g., combination of the potential consequences for each incident with the incident frequency and summing over all events), and
6. evaluation of the risk (e.g., identify the major sources of risk and determination if there are cost-effective process or modifications to reduce risk).

NanoRisk itself does not generate specific guidance regarding quantitative estimation of risk associated with ENMs and does not provide a stand-alone methodology for integrating quantitative and qualitative information related to risk potential. However, NanoRisk does document a series of possible risk management decisions that should be addressed and provides recommendation on how to document specific risk management options.

(c) The **US EPA’s Comprehensive Environmental Assessment (CEA) Framework** provides a high-level set of recommendations for approaching the subject of assessing the potential health and environmental impacts of nanomaterials [42]. CEA recommends following a traditional risk assessment process [18], but stresses the need for considering the complete product lifecycle, transport and transformation in the environment, and exposure potential or absorbed dose (by all exposure pathways), in addition to impact assessment. CEA recommends the construction of an information system that considers both an expert domain knowledge (including via meta-analysis) and utilization of various LCA methods, cost-benefit analysis, and decision science methods, while engaging stakeholders in the CEA process. CEA was evaluated via a case study [43] in which stakeholder engagement (expert elicitation) served to collect information about the risk potential of using multi-walled carbon nanotubes (MWCNTs) in flame-retardant coatings in upholstery textiles. Expert opinions were elicited, via a web-based tool (“CEAWeb”) [43] to prioritize the range of needed studies on MWCNT release across the product life cycle and human exposure or health impacts, which included, for example, defining/quantifying exposure scenarios, effects of MWCNT functionalization, developing techniques to quantify MWCNTs in air and other media, and estimation of safety thresholds [43]. CEA case studies were also documented for Nano Ag and Nano TiO_2_, in which information gaps were assessed to identify future research needs and priorities [72]. Overall, although CEA provides a useful roadmap for evaluating the potential impacts of ENMs, this “framework” is essentially a guidance document that falls short of providing or recommending specific quantitative methodologies for the integration and analysis of information/data to assess the risk potential of ENMs.

(d) **NanoHAZ** is an approach developed specifically for assessing the potential ecological risks associated with ENMs (including human risks associated with exposure to ENMs through environmental media) in Ireland [44]. NanoHAZ is based on comparison of estimated ENMs concentrations with existing regulatory limits for specific ENMs or their chemical building blocks [44]. The approach relies on probabilistic material flow analysis (MFA) with heuristics or assumptions based on empirical knowledge regarding the potential ENM exposure concentrations in the various media. The estimated exposure level in a given media (primarily air and water) for the target ENM is then compared to a bench-mark exposure concentration or critical concentration at which a specific effect is observed (as determined from in vivo toxicological studies) for the target receptors (human or ecological). One limitation of the NanoHAZ approach is the paucity of regulatory limits for ENMs [44]. As a result, the initial reported application of the approach, which focused on metal and metal oxide ENMs, utilized regulatory limits on exposure concentrations for dissolved metals or chemical building blocks of the ENMs as surrogates for the ENMs themselves [44]. Specific NanoHAZ case studies were reported for nano TiO_2_ in paints, nano Ag as an antimicrobial agent in food packaging, and nano CeO_2_ as a fuel additive. It was concluded that the level of concern regarding inhalation exposure to airborne nano CeO_2_, associated with its use as a fuel additive, was higher relative to concern regarding air releases of nano Ag and nano TiO_2_. The level of concern for nano TiO_2_ was considered moderate given its relative high score of potential exposure in drinking and surface (relative to nano-Ag and nano-CeO_2_), and low relative score of hazard (e.g., ecotoxicological and toxicological effects) compared to nano-Ag. Finally, nano-Ag as an antimicrobial agent in food packaging was considered of low concern given its low score for potential exposure (lower release expected in water compared to nano-TiO_2_) despite its moderate and high scores for ecotoxicological and toxicological effects.

Overall, the application of NanoHAZ can be useful if information is available regarding environmental releases of ENMs and their potential toxic effects are known or can be predicted from suitable models. As described in the available case study [44] NanoHAZ can serve to compare and rank ENMs with regard to their potential exposure and hazard.

(e) The **Nanomaterial risk-screening tool (NRST)** was developed on the basis of expert opinions compiled at a nanotechnology workshop that focused on assessing the importance of various factors that may affect hazard, exposure and risk associated with ENMs. The framework was formulated as an excel spreadsheet in which the analyst can select qualitative “risk ratings” (scale of 1–5, where 1 represents the lowest concern) [45]. The hazard rating is then calculated as the linear aggregation (using weight factors) of scores assigned to each contributing ENM physicochemical attribute (e.g., ENM chemical composition, crystallinity, average size, aspect ratio, surface area and charge, reactivity, solubility, hydrophobicity, agglomeration and sorption tendency) and contributing ENM hazard indicators (e.g., ENM potential for inducing ROS and mobility through cells). The exposure rating is determined based on aggregation of individual scores assigned to factors linked to environmental and human exposure potential during product manufacturing, use and end-of-life. These factors include product characteristics (e.g., content of ENM in product and form, product type) and exposure indicators (e.g., ENM environmental release potential, frequency and duration of exposure, number of exposed individuals). The aggregation of scores follows an assumption of linear additivity with assumed weight factors. It is noted that the aggregation approach does not provide for the establishment of bi-directional cause-effect relationship pathways. Therefore, one cannot directly ascertain the reliability of the obtained ranking relative to the existing quantitative body of evidence. Overall, however, the approach is a useful first step in organizing information and opinions to arrive at an initial ranking of concerns as being high, medium or low.

(f) **Engineered Nanoparticles – Review of Health and Environmental Safety: Human Health and Ecological Risk Assessment (ENRHES RA)** is a framework developed as part of the European Union project “Engineered Nanoparticles: Review of Health and Environmental Safety (ENRHES)” [46]. The goal of ENRHES is to facilitate estimation of ecological and human health impacts of ENMs and identification of data gaps for regulatory risk assessment under the European REACH (Registration, Evaluation, Authorization and Restriction of Chemicals) guidelines [46]. While the focus on human risk assessment presented in the published case studies [46] is not occupational, the exposure profiles reviewed included human exposure via manufacturing, consumer products and contact with the environment. The first step of the analysis process entails hazard identification (e.g., obtaining indicative no effect concentrations (INEC) for ecological receptors and indicative of no effect levels (INELs) for human population from published data) [73–76]. The second step consists of exposure assessment, performed on the basis of evaluating the occupational exposure for human receptors reported in the literature for the target ENM(s). Environmental exposures are qualitatively estimated using the expected or known ENM presence in environmental compartments based on estimates obtained from material flow analysis (MFA) [77]. It is noted that the MFA data, incorporated into the ENRHES RA, are not based on fundamental modeling of multimedia fate and transport. Thus, it is possible for mass balance inconsistencies to arise and violations of constraints imposed by intermedia transport mechanisms. The third step consists of risk characterization for human and ecological receptors. For human risk characterization, the measured and/or monitored occupational exposure concentrations were compared with the INELs, whereas for ecological risk assessment the modeled ENMs concentrations (e.g., orders of magnitude ng/L, µg/L, µg/m^3^) were compared with the INEC values.

ENRHES RA was demonstrated in a case study [46] exploring the potential human risk of four ENMs (nano-Ag, nano-TiO_2_, nano-ZnO, fullerenes and carbon nanotubes (CNTs). The analysis revealed that the INELs of fullerenes, nano-Ag and nano-TiO_2_ are lower than most of the reported occupational exposure concentrations for these materials. It was also suggested that the exposure concentrations of concern, for ecological receptors, are likely to be due to release of the ENMs into water in the following decreasing level of concern: ZnO >> Nano-Ag >> Nano-TiO_2_ > (MWCNT=C_60_) [46].

In summary, the application of ENRHES RA framework for ENMs is particularly useful as a roadmap for the REACH process. While the approach provides a conceptual based description of the analysis process, as illustrated by case studies, application of the ENRHES RA framework is at present limited by the availability of exposure and hazard information for the target ENMs.

(g) **Risk Quantification Based on Probabilistic Mass Flow Analysis (PMFA risk Quantification)** was proposed as a basis for risk-based classification system of ENMs present in water and soils with the goal of quantifying the probability of environmental risks [47,78]. The approach relies on a probabilistic material-flow analysis (PMFA) [78] to estimate the releases of ENMs to the environment on the basis of available data and expert judgement regarding production, use and disposal, along with heuristic and empirical assumptions to arrive at potential exposure concentrations in various media [79]. Published toxicity data (e.g., terrestrial and aquatic species tested for no observed effect concentrations, lowest observed effect concentrations and lethal concentrations for a 50% of the population) are used as inputs. The above compiled information is then used to build species sensitivity distribution (SSD) models [80]. SSD models have also been used by the US EPA to summarize evidence for stressor-response relationships obtained from laboratory studies [81]. In such an approach, the risk probability metric is defined as the product of the probability distribution of the predicted environmental concentrations and the probability that one or more organisms would be negatively impacted as a function of environmental concentration. In such analysis, zero percent risk indicates that all predicted environmental concentrations are lower than the lowest limit of the probabilistic SSD, and a 100% risk means that all predicted environmental concentrations overlap with the probabilistic SSD. Using this approach, Gottschalk et al. [47] evaluated the relative environmental risk posed by selected ENMs in Switzerland. It was reported that the highest risk, due to releases from sewage treatment plants, was associated with nano-Ag (40% overlap of the modeled environmental concentrations with the SSD for aquatic species), followed by nano-TiO_2_ (19% overlap) and nano-ZnO (1% overlap). With regard to ENMs found in surface water, nano-Ag was reported to present a higher risk (1% overlap) than nano-TiO_2_ (<0.1% overlap). In contrast, the authors concluded that there was no measurable risk related to CNTs and fullerenes in any of the studied environmental compartments (e.g., water and soil) [47].

The PMFA framework is useful if quantitative data/information are available to construct the SSD and to estimate environmental concentrations. However, the application of the PMFA framework also requires expertise to conduct the analysis and reliance on expert judgement in estimating exposure concentrations and the SSD.

(h) **Forecasting of the Impacts of Nanomaterials in the Environment (FINE) Based on Bayesian Networks (BN)** is an approach proposed to formally incorporate expert judgments to address data gaps and provide a probabilistic measure of potential environmental impacts of ENMs [48]. The above approach is suitable for both incremental learning and propagation of uncertainties [82]. The initial demonstration of this method was an assessment of the environmental impacts of nano-Ag in water and sediment [48]. BN were used to integrate quantitative and qualitative information, address data gaps, quantify uncertainties, and provide bidirectional causal relationships. Briefly, the BN approach consists of two main parts: 1) development of the network structure (nodes and their connectivity), and 2) determination of baseline parameters for each node in the form of conditional probability tables (CPTs). In the test study reported for nano-AG [48], the BN structure was developed on the basis of expert elicitation and consisted of nodes that were grouped into three categories: i) media parameters (e.g., temperature, pH, presence of organic matter), ENM properties (e.g., ENM coatings, zeta potential, fractal dimension, ENM diameter) and ENM transformations (e.g., ENM aggregation potential, attachment efficiency, biodegradation, dissolution and deposition); ii) exposure potential (e.g., ENM concentration entering system, concentration in sediment, water and dissolved concentration), and iii) hazard potential (e.g., bioavailability potential, biouptake, effects on biomass/mortality, effects on the ecosystem, such as decomposition, methanogenesis, eutrophication). In the case study reported by Money et al. [48], the CPT for the different nodes and individual variables (input values, units, ranges and categories) were established based on expert judgment, and the BN was applied to estimate ecological risks (e.g., probability distribution of risk being <1 or >=1) posed by nano-Ag particles present in the aquatic environment. The case study suggested that the greatest potential risk is expected when nano-Ag is accumulated in sediments rather than in water [48]. Given that the FINE BN framework was tailored specifically for nano-Ag in water, its applications is relevant to the aquatic environment. However, it is stressed that FINE BN can be tailored to different ENMs, and various environmental media, provided that the BN design includes the causal relationships governing the various aspects of the environmental fate and transport and toxicity behavior of the classes of ENMs under consideration. The FINE BN framework can be particularly useful for integrating quantitative and qualitative information and for enabling periodic updates (i.e., as new data becomes available) via incremental learning.

(i) **Life Cycle Risk Analysis for Nanomaterials (Nano LCRA)** is a screening approach developed with the intent of identifying potential risks and data gaps over a nanoproduct’s life cycle [49,55]. Nano LCRA incorporates relevant data through the life cycle of the target ENM with the intent of informing risk management practices and prioritizing research strategies. The analysis consists of the following steps:

1. Description of the life cycle of the product;
2. Identification of the materials and assessment of the potential hazards in each life cycle stage;
3. Exposure assessment for each life cycle stage;
4. Identification of the life cycle sages in which exposure may occur;
5. Evaluation of potential human and nonhuman toxicity at the key life cycle stages;
6. Analysis of risk potential for selected life cycle stages;
7. Identification of key uncertainties and data gaps and communication of findings;
8. Development of mitigation/risk-management strategies;
9. Gathering additional information (e.g., data that might have been identified as missing from the assessment);
10. Evaluating the efficiency of the developed risk management strategies and identifying the next set of priorities (e.g., identify newly available data to update mitigation/risk-management strategies) [49].

The Nano LCRA framework was applied to assess the potential risks of using cellulose nanomaterials (CNs) as substitutes for resource-intensive materials, such as plastics, including those used in commercial applications (e.g., packaging, composite polymers, paints, cosmetics, water and air filtration, and recyclable electronics), and to identify data gaps [50]. Case study results indicated that the highest priority for the development of new data is the need for information/data regarding occupational inhalation exposure associated with handling CNs as a dry powder. The authors also concluded that there is a significant knowledge gap regarding the toxicity of CNs used in consumer products, such as packaging.

The Nano LCRA framework appears to be useful for qualitative analysis. However, the available studies have not incorporated a method for integration of quantitative data (e.g., release amounts of ENMs to the environment, predicted/calculated environmental concentrations, toxicity thresholds, etc.) Evaluation of the reported LCRA case study suggests that use of the Nano LCRA framework would require extensive data collection and analysis expertise throughout the various steps.

### Frameworks for risk characterization in occupational settings

In general, the proposed frameworks to characterize risks in occupational settings reflect efforts to adapt existing environmental RA approaches for conventional chemicals to develop and implement effective risk management (RM) guidance for addressing the risks of occupational exposures to ENMs [51]. Four different frameworks for characterizing the occupational risks of ENMs were evaluated: (a) the Risk-Based Classification for Occupational Exposure Control (“Risk-Based OEL”) approach, (b) the Risk Classification Based on an Industry Insurance Protocol (RCIP) approach, (c) CB Nanotool, and (d) the Web-Based Tool for Risk Prioritization of Airborne Manufactured Nano Objects (“Stoffenmanager Nano”).

(a) The **Risk-Based Classification for Occupational Exposure Control (“Risk-Based OEC”)** approach was proposed to facilitate the development of occupational exposure levels (OELs) to improve risk management (reduce workers’ exposure) in the workplace [51,83]. In the Risk-Based OEC approach, hazard of ENMs are evaluated and risk estimates (e.g., % of excess risk) are developed. In cases where limited hazard data are available for the ENMs, hazard data for reference (benchmark) materials are used. Reference materials are selected based on whether they exhibit similar chemical/materials properties and similar modes of action (MoA) to the ENM of interest (e.g., for nano-TiO_2_, data for fine and ultrafine TiO_2_ were used). Examples of modes of action include ROS formation, genotoxicity, or interference with specific cellular functions. The risk potential for exposure to the new ENM(s) in occupational settings via inhalation is then systematically compared with those of benchmark material(s) in the same MoA class. For example, Kuempel et al. [51] used the approach to assess the risk potential of exposure to a variety of airborne particles, including both fine and ultrafine materials. The following standard risk assessment process steps were followed [18]:

1. Identifying the relevant animal model, dose metric, and disease response;
2. Modeling the animal dose–response relationship and estimate the critical effect level (e.g., benchmark dose);
3. Extrapolating the animal critical effect level estimates to humans by adjusting for factors that influence the deposited or retained lung dose in each species, assuming equal response at equivalent dose;
4. Estimating airborne exposures (8 h time weighted average (TWA)) that would result in the human-equivalent dose.

The authors then calculated the 1/1,000 excess risk of lung cancer based on animal-to human extrapolation of benchmark dose estimates (“BMD” is a dose associated with a specified increase in the probability of a given response known as the “benchmark response” (BMR)) using a multistage cancer model and the US EPA’s BMD software [84]. Four risk categories were established in the above case study, for ENMs and fine-sized particles in air based on information derived from previously reviewed control approaches [85]:

1. Low Risk bin/category aimed at dusts at an airborne concentration range >1 mg/m^3^ and where exposure can be controlled with general ventilation measures (e.g., fine-sized particles TiO_2_ and MoO_3_ at concentrations in the range of 1,000–4,000 µg/m^3^ TWA;
2. Moderate Risk bin for dusts at an airborne concentration range (0.1–1 mg/m^3^), which can be controlled with local exhaust ventilation measures; (e.g., carbon black, diesel exhaust particulate (DEP), and ultrafine TiO_2_ at TWA airborne concentration (90–250 µg/m^3^);
3. High Risk categories for dusts at an airborne concentration range (0.01–0.1 mg/m^3^), which can be controlled through ventilated enclosures; (e.g., fine particles of NiO and soluble CoSO_4_ at TWA 20–30 µg/m^3^);
4. Very High Risk (dusts at airborne concentrations 0.001–0.01 mg/m^3^), which can be controlled with containment systems (e.g., fine particles Ni_3_S_2_ and GaAs at TWA 4–5 µg/m^3^).

The above Risk-Based OEC is useful for grouping inhalable ENMs in occupational settings on the basis of workers’ exposure to ENMs or their ultrafine counterparts. However, considerations of the latter also require adequate characterization and toxicity/hazard data.

(b) The **Risk Classification Based on an Industry Insurance Protocol (RCIP)** was designed to compare risks associated with specific steps in the manufacturing of ENMs (as opposed to overall occupational risk) with those of traditional chemicals used in current activities such as petroleum refining, polyethylene production, and synthetic pharmaceutical production [52]. This framework follows two major parts. The first part involves data collection for each of the steps of a particular manufacturing/synthesis process including, inventory of input or constituent materials, output materials, waste streams, and physical conditions of the manufacturing processes (e.g., temperature, pressure and enthalpy, if available, or representative synthesis methods including a full description of the processes in form of flowcharts). For each constituent material the data to be collected include: toxicity values (e.g., LC_50_ and/or LD_50_), water solubility, octanol–water partition coefficient, flammability, expected emissions, molecular weight, and photolysis and degradation rates (e.g., photolysis and degradation rates are considered to predict mobility of a material). In the second part of the framework an actuarial tool (“XL tool”) is used to assign and tabulate risk scores to the operating conditions of the chemical processes, as well as to hazardous properties and toxicity values of the constituent materials.

The XL tool follows a protocol that is routinely used by industry to calculate insurance premiums. In the RCIP framework, the XL analysis involves a series of arithmetic operations to calculate additive scores for the “risk” posed by a specific process, the “risk” posed by the hazard/toxicity of the constituent materials, and the “risk” posed by the amount of the material emitted. After additive scores for the individual parameters inventoried in step (1) are obtained, an aggregated score is calculated (e.g., the sum of the “risk” posed by the process, the “risk” posed by the hazard/toxicity of the constituent materials and the “risk” posed by the amount of the material emitted). The aggregated score is calculated considering two scenarios: a) normal conditions of operation (e.g., assuming that none of the constituent materials are mobile and that photolysis and degradation do not occur); and b) an accident scenario (to account for what might occur if there was an accidental emission resulting in mobility of the constituent materials/chemicals); photolysis and degradation rates are also considered along with process conditions (e.g., temperature, pressure and heat transfer). These two aggregated scores, for the normal conditions and accident scenarios are then added and normalized with respect to the highest score to yield an overall score, which is referred to as the latent risk score.

The above approach was demonstrated by Robichaud et al. [52], for a case study that considered representative synthetic processes for selected ENMs (C_60_, single-walled carbon nanotubes (SWCNT), multi-walled carbon nanotubes (MWCNT), cadmium selenide (CdSe) and zinc selenide (ZnSe) quantum dots, carbon black, aluminum and silver nanoparticles (nano-Al and nano-Ag)) that were compared to synthetic processes for traditional chemicals (petroleum refining, polyethylene production, and synthetic pharmaceutical production). The analysis suggested that the manufacturing of the ENMs studied might present lower risks than for the chemicals listed above.

(c) The Control Banding (**CB) Nanotool** [53] is an approach developed with the intent of identifying/prioritizing health risks in the workplace in order to assist in the implementation of exposure controls [53]. Control banding is a term originated from the field of industrial hygiene [53] and represents a qualitative approach to assessing risks associated with chemicals with the goal of developing suitable control measures (e.g., via personal protective equipment, administrative or engineering controls). In CB Nanotool, categories or ‘‘bands’’ are established for health hazards of ENMs, which are then combined with exposure scenarios for the target ENMs, to determine recommended levels of control. An advantage of this approach is that it can be used even in the absence of toxicity data for the specific ENM of interest. The above is regarded as a practical approach in the field of ENMs occupational risk management, given the need to provide recommendations for control measures in the absence of complete hazard profiles for the rapidly growing number of new ENMs [53].

The CB Nanotool [53] was designed specifically for inhaled ENMs to determine the level of risk of operations carried out in research laboratories. In this approach, the risk level band is assigned based on a matrix that combines two scores, one for severity (e.g., degree of biological response elicited by the ENM exposure via inhalation or presence in the bloodstream) and one for probability (e.g., the extent to which employees may be potentially exposed to ENMs throughout the handling processes). The severity score is calculated by adding individual scores (e.g., scores assigned via the guidelines recommended by the authors) for physicochemical properties of the ENM (e.g., surface chemistry, particle shape, particle diameter, solubility) and evidence of toxicity (e.g., reproductive, carcinogenic, mutagenicity, dermal and acute toxicity) available for the ENM and for the ENM bulk counterpart (main chemical substance in the composition of the ENM). A probability score is calculated by adding individual scores assigned to the estimated amount of handled ENM (i.e., by the worker), dustiness/mistiness, number of employees with similar exposure, and duration of operation. Similar to the severity score, the proposed approach provides guidelines for assignment of values to each of the parameters of the probability score. The final product is presented as a combined score of the severity and probability parameters which are assigned to control bands. The combined score or assignation to a control band (e.g., RL) is done qualitatively via a matrix in which the severity scores of low, medium, high and very high grouped by category as rows, while the probability scores of extremely high, less likely, likely and probable grouped by category as columns. For example, the box assigned to the combination of the highest probability score with the highest score of severity will result in the highest band of recommended control measures (e.g., Risk Level 4 (RL 4), “seek specialist advice”). As the combination of scores decreases in value, the assigned bands correspond to lower recommended control measures. An EXCEL sheet for use with the above approach was reported by Paik et al. [53] and later evaluated by Zalk et al. [86].

The CB Nanotool represents a framework that is useful for identifying potential control measures for workers’ protection. Its utility, however, is predicated on the availability of information on the various activities/steps (e.g., handling ENMs in powder form) involved in the ENM manufacturing process, as well as the hazards posed by the ENMs.

(d) The **Web-Based Tool for Risk Prioritization of Airborne Manufactured Nano Objects (“Stoffenmanager Nano”)** [54] is a framework based on control banding, similar to the CB Nanotool. The Stoffenmanager Nano approach aims at identifying control measures to reduce the likelihood of inhalation exposure in occupational settings. This framework requires both exposure and industrial process information (e.g., point or fugitive emissions during production, handling powdered ENMs, dispersion of ENMs and activities resulting in ENM release, such as sanding of surfaces) and hazard identification parameters (e.g., solubility of ENMs, nanofiber shape, toxicological data of the ENM or parent material) as inputs. The approach is divided into two steps: 1) an assignment of a hazard category for the ENM and 2) an assignment of an exposure category for the industrial process.

In the first step, one of five hazard categories (A–E, where A and E represent the lowest and highest hazards, respectively) is assigned based on available data. For example, hazard classification can be made based on the water solubility of the ENM (i.e., high water solubility would suggests lower hazard as an ENM and thus such ENM would be in category (A) or based on persistence of nanofibers (where persistent nanofibers would result in a high hazard category of E); other ENM hazard data can also be taken into account at this stage (e.g., a band (B) is given to those ENM considered as irritant, a band (C) is given to an irritant that also causes burns). A table built based on expert elicitation with pre-assigned hazard bands is provided in the Stoffenmanager Nano tool for selected ENMs (i.e., C_60_, carbon black, Ag, Fe, Au, Pb, La, TiN, TiO_2_, CeO_2_, ZnO and others such as nanoclay and polystyrene) [54]. In general, however, the hazard band assignment in Stoffenmanager Nano is dependent upon the assessor’s judgement and/or the guidelines/thresholds provided by the tool developers.

In the second step, the user has to select an exposure band value (range of 1 to 4, where 1 and 4 represent the lowest and highest exposure, respectively). The exposure band is assigned via scores (termed multipliers in the Stoffenmanager Nano tool) which take on numerical values proposed by the authors based on previously published data and or expert elicitation [54]. The scores provided by Stoffenmanager Nano tool are for various factors that influence exposure (e.g., substance emission potential, handling/activity emission potential, localized controls, segregation, dilution/dispersion, personal behavior, separation/personal enclosure, surface contamination, and respiratory protective equipment) for the industrial process/setting under consideration. Scores are then assigned to 4 bands depending on their value range. Once the hazard and exposure bands are assigned, a matrix is built that qualitatively combines the hazard (columns A–E) and exposure bands (rows 1–4) to yield the priority band (scale of 1–3, where 1, 2 and 3 are for high medium and low priorities, respectively, for exposure control). Following the above approach, for example, the highest priority (band 3) is associated with ENMs having both the highest hazard and highest exposure bands.

The Stoffenmanager Nano framework is particularly suited to situations where the industrial processes involving ENMs are known and where there is potential for inhalation exposure. Application of Stoffenmanager Nano allows the user to rank/prioritize ENMs based on potential worker exposure, which can be useful in situations where decisions must be made with limited data.

### Evaluation of the different risk assessment frameworks

To assess the utility of available risk assessment frameworks for ENMs described above, the following questions were posed:

1. What is the intent of the framework and who are the potential users/decision makers for which the framework is designed?
2. What is the level of resolution/type of results needed by the potential decision makers to be able to make risk management decisions about the target ENMs?
3. What is the level of expertise that the user must possess to conduct the analysis using the framework?

When addressing the first question, each framework was evaluated to determine if it addresses one or more of the six different decision-making scenarios described in the Methods section and in Table 2. Existing frameworks, which are most suitable for each of the posed decision-making scenario were also identified (Table 2). Lastly, for each decision-making scenario the critical needs that are not met by any of the existing frameworks were identified.

**Table 2:** Decision needs and recommended ENMs relevant risk assessment frameworks for selected regulatory decision-making scenarios.

Scenario	Example and desired output of analysis	Potential framework for use/ currently available frameworks

Scenario I: Company deciding whether to control exposure to workers during manufacturing or processing of ENMs.	A company is producing a new ENM and needs to identify the controls necessary to protect their workers.	Swiss Precautionary Matrix; DuPont NanoRisk [41]; Control Banding (CB Nanotool) [53]; Web-Based Tool for Risk Prioritization of Airborne Manufactured Nano Objects (Stoffenmanager Nano) [54].
	Internal risk management strategy including recommended engineering controls, administrative controls	
Scenario II: Regulatory body deciding whether to control exposure to workers during manufacturing or processing.	OSHA deciding whether to establish Occupational Exposure Limits (OEL)/Permissible Exposure Limits (PEL) for a specific class of ENMs.	Risk based classification for occupational exposure control (Risk based OEL) [51], SPM [31].
	Evidence based recommendations or requirements for allowed exposure.	
Scenario III: Company deciding whether risk associated with producing a nanoparticle or nano-enabled product is manageable.	Company needs to assess the potential impacts of the production of a nano-enabled product and how to manage risks if any.	Web-Based Tool for Risk Prioritization of Airborne Manufactured Nano Objects (Stoffenmanager Nano) [54]; Risk classification based on an Industry Insurance Protocol (RCIP) [52]; An Adaptive Screening-Level Life Cycle Risk-Assessment Framework for Nanotechnology (Nano LCRA) [50,55].
	Risk assessment of a particular ENM and risk management strategy.	
Scenario IV: Company deciding which nanoparticle or nano-enabled product poses less risk than alternatives for a particular application.	Company interested in a precautionary approach for safe-by-design applications.	Multi-criteria Decision Analysis (MCDA) [34], Life Cycle Analysis [40], FINE (Forecasting the Impacts of Nanomaterials in the Environment) based on Bayesian Networks [48], modified GreenScreen [52].
	Assessment or comparison of alternatives in terms of environmental impacts and technical performance.	
Scenario V: Regulatory body deciding whether to control environmental use, release, or emissions of an ENM.	US EPA deciding whether to issue a Significant New Use Rule (SNUR) under TSCA (Toxic Substances Control Act) for a particular type of ENM.	US EPA’s Comprehensive Environmental Analysis (CEA) [42]; Risk Assessment Framework for Assessing Metallic Nanomaterials of Environmental Concern (NanoHAZ) [87]; A risk quantification based on probabilistic flow modeling analysis (PMFA); Forecasting the Impacts of Nanomaterials in the Environment (FINE) based on Bayesian Networks [48]; Nano material risk-screening tool (NRST) [45].
	Substantial evidence to indicate that a specific ENM will present an unreasonable risk to people or the environment.	
Scenario VI: Regulatory body deciding whether to allow nanoparticles to be included in food, drugs, personal care products.	US FDA deciding whether to allow registration of a new nano-enabled product in food (whole food, dietary supplement, food ingredient or additive), medical devices, drugs or cosmetics.	NanoRiskCat [35]; Engineered Nanoparticles – Review of Health and Environmental Safety: Human health and Ecological Risk Assessment (ENRHES RA) [46]; DF4Nano [36].

Safety assessment for cosmetic products or a Risk Evaluation and Mitigation Strategy (REMS) for a new drug [30].

#### Suitable frameworks for Scenario I (“A company needs to decide whether to control exposure to workers during manufacturing or processing of ENMs”)

The most suitable existing frameworks for Scenario I are the Swiss Precautionary Matrix (SPM) [31], the DuPont NanoRisk [41], Control Banding Nanotool [53], and Stoffenmanager Nano [54]. Each one of these frameworks has different capabilities that companies can use to assess the need for controlling workers’ exposure to ENMs. For example, SPM can be used to identify hazards and/or the need for further actions in terms of risk management related to manufacturing processes of ENMs. SPM allows for rapid assessment of known/unknown information (first tier assessment). The questions posed in SPM are mostly qualitative and designed to determine whether or not the user is dealing with a material that is considered to be classified as “nano”. SPM was developed by the Swiss Federal Office of the Environment and the Federal Office of Public Health. Therefore, SPM includes pertinent regulatory definitions for nanomaterials (relevant for Switzerland) and provides useful guidelines to industry users wishing to comply with environmental health and safety regulations. The downside is that this framework does not include a detailed analysis of industrial processes with respect to parameters related to worker safety (e.g., the number of employees exposed, frequency of exposure, control measures already in place). Another limitation is that SPM does not provide the analyst with specific recommendations for implementation of industrial hygiene controls.

The DuPont NanoRisk framework can address the specific needs for a risk management strategy through ENM life cycle profiles. This framework is suitable for decision making related to controlling worker exposures because the DuPont NanoRisk framework requires the analyst to provide lifecycle, exposure and hazard profiles for the material of interest. The challenge with the DuPont NanoRisk framework, however, is that it requires input of a significant body of information in addition to conducting a chemical process risk assessment (CPQRA).

Banding approaches such as the Control Banding Nanotool and Stoffenmanager Nano are useful for classifying ENMs and establishing risk management needs (e.g., reducing working exposure via engineering controls, personal protection equipment and other measures). Because CB Nanotool involves the identification and quantification of extensive characteristics of the industrial processes (e.g., number of workers potential exposed, frequency of exposure, concentrations that the workers could be exposed to), it allows the user to tailor protective measures to the company’s needs. Two primary disadvantages of CB Nanotool are that it requires significant user data input, and that the procedure or the decision regarding the “bands” is highly dependent on the knowledge/expertise of the assessor. Stoffenmanager Nano also requires extensive input information input by the analyst. However, Stoffenmanager Nano takes into consideration the use of personal protective equipment (PPE) and current industrial hygiene (IH) practices; hence, this approach is useful for reviewing current practices and for leading the analyst to identify possible needs for modifying current practices. Another advantage is that Stoffenmanager Nano is accessible as a web-based tool.

#### Suitable frameworks for Scenario II (“Regulatory body that has to decide whether to control exposure to workers during ENM manufacturing or processing”)

In Scenario II, the stakeholders wish to establish an evidence based exposure limit (e.g., OSHA established safe exposure level setting) for an ENM of concern. Of the existing frameworks, the Risk-based OEL framework proposed [51,83] is most suitable for this scenario, particularly in cases where a benchmark dose for a reference material (e.g., for a corresponding bulk material or ultrafine material) is available. The risk-based OEL framework also offers the advantage of identifying specific/minimum data required for conducting an assessment, which allows users to prioritize future research and data collection. However, the applicability of this framework is limited to assessment of ENMs for which well characterized ultrafine counterparts exist; therefore, the approach may have limited utility for next generation ENMs for which well-characterized reference materials are not available. Additionally, given the SPM parameters (potential effect (W), potential exposure of humans or environmental release (E), and available information on the material’s life cycle) [31] this framework could be useful for the regulatory agency to identify potential ENMs of concern, hence preventing workers’ exposure.

#### Suitable frameworks for Scenario III (“A company that needs to decide if the risk(s) associated with producing a nanoparticle or nano-enabled product is manageable”)

In Scenario III, the stakeholders are individuals working for a company that needs to ascertain whether the risks associated with producing a nanoparticle or nano-enabled product can be reasonably managed. The most suitable frameworks for this scenario are the: Web-Based Tool for Risk Prioritization of Airborne Manufactured Nano Objects (Stoffenmanager Nano), Risk Classification based on an Industry Insurance Protocol (RCIP), and Life Cycle Risk Analysis (Nano LCRA). Each of these frameworks allows the analyst to assess impacts related to production of certain ENMs and to identify risk management/reduction strategies. Stoffenmanager Nano is suitable for this scenario as it allows one to design risk reduction/management strategies for each of the “risk bands”, which can then be applied to any ENM that meets the classification criteria for each risk band. Stoffenmanager Nano requires detailed information about both the ENM and the associated industrial handling operations. The above is needed to arrive at informative strategies to manage risks associated with the material. However, it is important to note that the above framework only provides a mechanism for qualitative assessment and suggestions of control measures of occupational risks, and risks related to potential releases to the environment that might occur during manufacturing.

The risk classification approach based on an insurance protocol framework is also suitable for Scenario III since it considers an “incident/accidental release scenario”. In this framework, a measure of the overall risk is calculated and the potentials for accidents are considered. This framework provides a detailed protocol, with the pertinent mathematical expressions, to calculate aggregate scores for parameters that affect risk (i.e., hazard and exposure). One limitation of this framework is that the risks associated with an ENM are calculated based on emissions, exposure potential and hazards of the chemicals involved in the synthesis of ENMs, not those for the actual ENM itself. Admittedly, the above limitation could also be perceived as an advantage if there is limited data for industry to assess the target ENM. Another limitation of the RCIP framework is that it does not address the development of risk management strategies.

Whereas both the Stoffenmanager Nano and RCIP frameworks focus primarily on risks related to manufacturing/synthesis of ENMs, NanoLCRA takes into account the potential risks attributed to the ENM throughout its lifecycle. A shortcoming of the NanoLCRA framework is that it does not provide a specific methodology for quantifying risk (e.g., steps for aggregation of scores, guideline tables to assign exposure/risk bands, or mathematical equations to derive reference values, and benchmark doses). Moreover, the framework relies entirely on an expert evaluation of the available information.

#### Suitable frameworks for Scenario IV (“A company that needs to decide as to which nanoparticle or nano-enabled product poses less risk than alternatives for a particular application”)

In Scenario IV**,** the stakeholders are individuals representing a company that desires to identify the safest ENM for a particular application. The most suitable existing frameworks are: Multi-Criteria Decision Analysis (MCDA), Life Cycle Analysis (LCA), BN FINE and modified GreenScreen. MCDA [34] is appropriate for Scenario IV because it allows comparison among alternatives. For example, MCDA was demonstrated for ranking the relative risk potential of a set of ENMs based on hazard related properties (e.g., agglomeration, potential to form ROS, reactivity, etc.) [34]. MCDA provides a framework for assessing properties related to hazard and, in doing so, allows the analyst to identify critical properties that could be modified to develop safer ENMs. The disadvantages of applying MCDA to ENMs, at least as is currently proposed in the literature [32,34,60], are that the approach relies primarily on expert judgment and that MCDA does not consider causal relationships (e.g., relating a specific ENM property to an adverse outcome).

LCA [40] is also suitable for companies that need to consider Scenario IV. This is because LCA provides a framework for assessing environmental impacts throughout the ENM life cycle (synthesis, use, and disposal). The use of LCA, however, requires a significant data (e.g., emission inventories for all chemicals involved in the manufacture of ENMs throughout their lifecycle).

BN FINE [48] is another useful framework for companies that need to address the above Scenario IV. Given that BN FINE involves the use of an influence diagram, which incorporates causal relationships between ENM properties and risk parameters, the approach can assist in identifying the relevant ENM properties that can be tailored to manufacture safe ENMs (i.e., “safer-by-design”) [88]. In the absence of quantitative data, expert judgement can be incorporated into the BN framework [48]; however, the framework developer must be able to identify the critical causal relationships (e.g., between ENM physicochemical properties, environmental conditions and risk outcomes). Although the BN approach is extremely powerful, construction of a BN based framework requires ENM specific data.

The modified GreenScreen [37,65] approach is another framework that can be partially suitable for Scenario IV as it allows analysts to perform rapid screening of potential hazards among a group of ENMs for which data are available. The scores provided by GreenScreen are designed to make recommendations regarding the need for additional information or for seeking safer ENMs. This framework is suitable for hazard assessment for Scenario IV but is not a substitute for risk assessment.

#### Suitable frameworks for Scenario V (“Regulatory body that needs to decide whether or not to control environmental use, release, or emissions of an ENM”)

Several existing frameworks are suitable for Scenario V, including US EPA’s own Comprehensive Environmental Assessment CEA [42–43], Nano HAZ [87], a risk quantification based on probabilistic flow modeling analysis (PMFA RQ) [47], BN FINE [48], and the Nanomaterial Risk-Screening Tool (NRST). CEA is useful for regulatory decision analysis (e.g., regarding issuance of Significant New Use Rule (SNUR) for a new ENM) because it can be used to systematically organize information. CEA provides a framework that allows decision makers to assemble and review data that are critical for determining whether a SNUR should be issued; such data includes, for example, the projected volume of manufacturing and processing, extent to which the novel ENM changes the exposure of human beings or the environment, and the anticipated manner and methods of manufacturing, processing, distribution in commerce, and disposal of a chemical substance. Advantages of the CEA framework include the provision of list/guidelines regarding the information needed for a comprehensive assessment, and the availability of a survey tool (“CEA web tool”) as a platform for eliciting expert information.

NanoHAZ and PMFA RQ are also suitable for use by regulators who are confronted with the need to reduce the potential environmental and health impacts of a specific ENM via restrictions on its environmental releases and use. NanoHAZ is specifically designed to provide qualitative estimations of risks for metallic ENMs in water treatment plants, via mass balance estimation of concentrations and with use of literature derived hazard data. Risk can also be estimated quantitatively in the PMFA RQ framework. It is noted that the PMFA RQ framework can also take into account local geographical and meteorological conditions and specific hazard data if these are available. It is emphasized that both NanoHAZ and PMFA RQ require the analyst to provide judgment as to whether the calculated risk is significant or unreasonable; such a request for information essentially implies that the analyst is knowledgeable regarding the implications of the various assumptions made by the frameworks’ developers.

Bayesian Network (BN) approaches like BN FINE can also be suitable for used under Scenario V. One advantage of this approach is that it allows incorporation of both quantitative and qualitative (including expert knowledge) data. BN offer the additional advantage of being able to conveniently refine/modify the BN as additional information becomes available (i.e., via incremental learning). Two additional advantages of BN FINE for regulators are that this framework can address ecological risks and can quantify uncertainties, thus assisting regulators in determining whether or not the calculated risk is significant/unreasonable.

Finally, the Nanomaterial Risk-Screening Tool (NRST) is a suitable framework for Scenario V because it takes into consideration both potential human and ecological risks associated with ENMs. However, given that this framework requires expert judgment regarding available information (and does not incorporate quantitative data), there may be a concern that potential bias could be introduced.

#### Suitable frameworks for Scenario VI (Regulatory body deciding whether to allow nanoparticles to be included in food, drugs, personal care products)

NanoRiskCat [35], ENRHES RA [46] and DF4Nano [36] are the most suitable frameworks available for Scenario VI because they focus on safety assessment for consumer products (e.g., new cosmetics or drugs applications), but each of them has significant limitations. NanoRiskCat is particularly useful for identifying potential exposure scenarios related to use of consumer products. Analysis via this framework, however, requires access to data regarding the form in which the ENM is present in the consumer product (e.g., as a spray, embedded in a solid film), as well knowledge of potential scenarios that can lead to ENM release to the environment. NanoRiskCat is also a suitable screening approach for identifying the need for more specific safety assessments. A major limitation, however, is that NanoRiskCat does not meet the requirements of REMS (Risk Evaluation and Mitigation Strategy); thus, it is less suitable for formal regulatory risk evaluation.

The DF4Nano framework can also be used to conduct a rapid assessment of human health hazards. If sufficient ENMs characterization data are available to allow a new ENM to be grouped with existing (better characterized) ENMs based on its properties, then DF4Nano can be used to classify the ENM risk potential in the absence of extensive toxicity data. One major limitation is that DF4Nano does not account for other product components or transformation of ENMs during product manufacturing.

The Human Health and Ecological Risk Assessment framework within the project “Engineered Nanoparticles – Review of Health and Environmental Safety” (ENRHES RA) can also be suitable under Scenario VI. One advantage of the ENRHES RA framework is that it can serve to estimate the risk potential of ENMs in consumer products, provided that data are available regarding ENMs properties and potential for release after incorporation into consumer products. A limitation of ENRHES RA is that it requires quantitative dose–response data and information regarding the potentially exposed population and exposure scenarios to be able to quantitatively assess the risks associated with a particular ENM.

## Conclusions

Over the last decade, a number of different analysis frameworks have been developed with the goal of providing evidence-based approaches to making practical decisions related to the potential risk associated with ENMs. The utility of these frameworks should be assessed based on the intent for making the decisions regarding the potential risk of ENMs and the level of decision making (i.e., who is and/or what is the authority of the decision maker?). Accordingly, the current review of existing frameworks for assessing the potential environmental and health impacts of ENMs evaluated the applicability of different frameworks based on six plausible decision scenarios. These scenarios were designed to describe the most common and pressing needs by critical stakeholders to arrive at decisions respecting the environmental health and safety of engineered nanomaterials (Table 2). For each of the explored decision scenarios, at least one existing framework was identified as being capable of partly meeting the needs of potential decision makers. Limitations and advantages of the different frameworks and associated available tools were then identified in relation to the needs for decision analysis.

Several of the existing frameworks were assessed to partially meet the needs of manufacturers and regulatory bodies seeking to identify measures for reducing or controlling workers’ exposure to ENMs during manufacture and other industrial activities (Scenarios I and II). These include the Swiss Precautionary Matrix, DuPont NanoRisk, Control Banding (CB Nanotool), and the Web-Based Tool for Risk Prioritization of Airborne Manufactured Nano Objects (Stoffenmanager Nano)). Each of these frameworks focuses on evaluating different activities that may lead to ENM exposure and incorporates hazard information to help the analyst develop and prioritize risk and exposure control measures for ENMs. However, because the above frameworks consider inhalation as the sole exposure pathway (with the exception of NanoRisk), they are of limited applicability to decision makers who wish to assess other exposure pathways (e.g., oral and dermal exposures).

Several frameworks that companies can use to assess or compare risks associated with production of nano-enabled products (Scenarios III and IV) are available; however, each of these frameworks requires either expert judgement, proprietary software packages, and/or extensive hazard data for the ENMs of interest. For instance, MCDA, LCA, BN FINE, Stoffenmanager Nano, RCIP, Nano LCRA frameworks all require access to extensive ENM toxicity and/or exposure data. MCDA and Nano LCRA also require expert judgment, while RCIP and BN FINE require significant expertise and use of external software packages.

Several frameworks have been designed to meet the need of decision makers (e.g., US EPA and US FDA) who wish to assess the potential impact of environmental releases of ENMs and safety of commercial products (Scenarios V and VI). However, each of these frameworks also has significant limitations. For example, the ability of regulatory decision makers to use CEA, NanoHAZ, PMFA, BN FINE, NRST, NanoRiskCat, ENRHES RA frameworks is limited to the types of hazard and exposure data that are currently available for ENMs. Because hazard and exposure data are typically only available for ENMs as manufactured, the above tools are not directly applicable for assessing risks posed by ENMs that have been transformed through their incorporation in nano-enabled products or their transformation in the environment. It is also noted that frameworks such as US EPA’s CEA and NRST do not include tools to integrate quantitative and qualitative information about hazard and exposure, and rely heavily on expert judgment to identify the data needed for the analysis.

Despite significant advances that have been made in the area of risk assessment associated with ENMs, the currently available frameworks do not provide a pragmatic, flexible and comprehensive approach that would meet the needs of all the critical categories of decision makers and decision scenarios. Given the varied decision analysis objectives it is not surprising that different risk assessment frameworks have been proposed at different levels of complexity, different data needs and with different outcome objectives. At present, the existing frameworks do not provide a convenient and transparent mechanism for integrating results from modeling tools with experimental and industry reported data. As a result, each of the existing frameworks is limited by the relatively incomplete exiting hazard and exposure data for ENMs. Given the rapid developments in nanotechnology, it would be highly desirable to develop an integrated framework that could provide an efficient mechanism for managing and integrating quantitative and qualitative information while also accounting for the impact of missing information as part of the analysis. Ideally, such a framework would also provide guidance to decision makers (in the absence of expert judgement) regarding the information needed to conduct decision analysis for specific scenarios that are of interest.

In closure, based on the present review of various risk assessment frameworks it is suggested that further research should focus on the development of integrative frameworks for assessing the risk potential of ENMs that: a) address the complexities of ENMs and their transformations, b) integrate quantitative and qualitative data, c) allow use of modeling tools to fill data gaps, d) minimize reliance on expert judgement, and e) enable quantification of uncertainties associated with the use of both quantitative and qualitative data/information. Such frameworks would not only be of practical use for decision makers in a variety of contexts, but would also provide evidence-based approaches for prioritizing future research and manufacturing of ENMs and related products in support of environmentally sustainable nanotechnology.

## Supporting Information

File 1 Additional details of reviewed frameworks, described in tabular format.
