# Supplementary material for: Needs and challenges for assessing the environmental impacts of engineered nanomaterials (ENMs)
Source: Beilstein J Nanotechnol. 2017 May 5;8:989–1014. doi: 10.3762/bjnano.8.101 (PMC5433198; doi:10.3762/bjnano.8.101)
Supplement: File 1 — Additional details of reviewed frameworks, described in tabular format. [file Beilstein_J_Nanotechnol-08-989-s001.pdf]

Supporting Information

for

Needs and challenges for assessing the environmental impacts of engineered nanomaterials (ENMs)

Michelle Romero-Franco[1,2], Hilary A. Godwin[1,2,3,4], Muhammad Bilal[1,3,4] and Yoram Cohen*[1,3,4,5]

Address: [1]University of California Center for Environmental Implications of Nanotechnology, University of California, Los Angeles 6522 CNSI Building, 570 Westwood Plaza Box 957227 Los Angeles, CA 90095-7227, USA, [2]Department of Environmental Health Sciences, Fielding School of Public Health, University of California, Los Angeles Box 951772; 56-070 CHS Los Angeles, California 90095, USA, [3]California Nano Systems Institute, University of California, Los Angeles 6522 CNSI Building, 570 Westwood Plaza Box 957227 Los Angeles, CA 90095-7227, USA, [4]UCLA Institute of the Environment and Sustainability, University of California, La Kretz Hall, Suite 300 Box 951496 Los Angeles, CA 90095-1496, USA and [5]Department of Chemical and Biomolecular Engineering, University of California, Los Angeles; 5531 Boelter Hall; Los Angeles, CA 90095-1592, USA

Email: Yoram Cohen - yoram@ucla.edu

* Corresponding author

Additional details of reviewed frameworks, described in tabular format

CONTENTS

Table S1: Analysis and Classification of Reviewed Hazard Identification Frameworks.

Table S2: Analysis and Classification of Reviewed Environmental Risk Assessment Frameworks.

Table S3: Analysis and Classification of Reviewed Occupational Risk Assessment Frameworks.

Table S1: Analysis and Classification of Reviewed Hazard Identification Frameworks.

Name of the framework and scope	Intrinsic characteristics	Input	Output	Address data gaps[(a)]	Software tools[(b)]
Swiss precautionary matrix [1]<br>*Application:* ENM containing products	Decision tree/questionnaire, based on published data, suitable for pre-screening of available ENMs data (e.g., physicochemical properties, hazard traits) and determination of further actions that may be needed (e.g., additional data, actions to control exposure)	Nano-relevance (*e.g.,* whether or not the analyzed ENM meets the definition under EU regulations), effects (*e.g.,* potential of the ENM for ROS formation, redox potential), potential for exposure (considering the maximum amounts released to the environment, frequency of exposure) and available information about the ENM life cycle (*e.g.,* source of the ENM).	Categories/classification of the hazard: group A - no need for review of (unspecified) risk management measures; group B - need for review of (unspecified) risk management measures or need for additional information.	No	Web application
Risk Classification System based on Multi Criteria Decision Analysis (MCDA risk classification) [2-4]<br>*Application:* ENMs	Outranking: given a set of alternatives that are compared in terms of performance for selected criteria designed via expert judgment. The user must assign scores (e.g., 1-4, 60-100, 5-200) that correspond to categories (e.g., low-very low, medium-low, medium, high, extremely high) for each pre-determined criterion (e.g., Agglomeration, Reactivity/charge, Critical function groups, Contaminant dissociation, Bioavailability potential, Bioaccumulation potential, Toxic potential, and Size) based on data or expert elicitation.	Data regarding specific properties of the ENM (*e.g.,* size, reactivity/charge, critical function groups) and transformation in the environment (*e.g.,* agglomeration, contaminant dissociation, bioavailability potential, bioaccumulation potential, toxicity). Data input can be from experimental studies or via expert judgment.	Categories/classification of the hazard (*e.g.,* toxicity): very low, low, medium, high, and extremely high.	Yes	MCDA general software (not specific for risk classification of ENMs)

Name of the framework and scope	Intrinsic characteristics	Input	Output	Address data gaps[(a)]	Software tools[(b)]
Hazard and exposure potential identification for ENMs in consumer products (NanoRiskCat) [5]<br>*Application*: ENM containing products	Decision tree/flowchart, where each of the steps is depicted in a flowchart, which requires a specific choice to be made by the analyst on the basis of available evidence.	Data regarding the potential hazard posed by the ENM based on: human health (*e.g.*, acute toxicity, germ cell mutagenicity, carcinogenicity, reproductive toxicity, specific target organ toxicity due to single or multiple exposures, skin corrosion and eye irritation) and environmental hazards (*e.g.*, ecotoxicity, persistence and bioaccumulation).	Categories/classification of the hazard: a color-coded system/visual representation of the potential hazards that includes a short description of the evidence (grey color assigned to insufficient data, green-low hazard potential, yellow-medium potential hazard and red-high hazard potential).	Yes	No
DF4Nano grouping [6]<br>*Application*: ENMs	Guidance for classification is given to the analyst in tables developed based on published data and reports. The analyst must choose the categories for evaluating the available information for comparison with values and thresholds provided in the framework.	Data regarding intrinsic ENM properties (water solubility, shape and aspect ratio, composition), system-related properties (use, release and exposure, uptake, biodistribution and biopersistence and bio-physical interactions) and toxic effects (endpoints from short term in vivo studies or toxicity evidence from in vitro studies) induced by the ENM.	Categories/classification of the ENMs: four main categories: 1) soluble ENMs, 2) biopersistent high-aspect ratio, 3) passive ENMs and 4) active ENMs. ENMs classified in 3 and 4 are considered to require a further analysis/risk assessment.	Yes	No
Modified GreenScreen [7]	Classification of hazard based on publicly available data and expert judgment. The analyst must assign a classification to the ENM data (e.g., low, medium, high concern) or indicate that classification is infeasible due to lack of data.	Data for the ENM includes following hazard endpoints: cancer, mutagenicity, reproductive toxicity, developmental toxicity, endocrine activity, acute mammalian toxicity, systemic toxicity (single and repeated exposure), neurotoxicity (single and repeated exposure), skin sensitization, respiratory sensitization, skin irritation, eye irritation, acute aquatic toxicity, chronic aquatic toxicity, in addition to environmental persistence, bioaccumulation, reactivity, and flammability. Data regarding ENM physicochemical properties and considering agglomeration and/or aggregation, chemical composition, purity, shape, surface area, surface charge; and surface chemistry (including composition and reactivity).	Benchmark (BM) categories (or recommendations) regarding ENM use are as follows: BM1 is for an ENM of very high concern as defined by U.S., Canadian and European regulatory bodies, BM2 and BM3 designate a material that can be continued to be used but safer substitutes are desirables as the nanomaterial may present human health concerns, BM4 is for a material that represents low hazards to humans and the environment, and a fifth category (BM-U) where information is insufficient to arrive at a classification.	No	Yes

(a) Capability of a framework to address data gaps: whether the framework includes a detailed methodology to obtain missing data (*e.g.*, consideration of expert judgment to address data gaps or modeling tools incorporated in the framework); (b) Availability of software tools specifically designed to conduct the analysis: whether software tools were developed or adapted to conduct the analysis. Specific tools included in this category include: spreadsheets (e.g., EXCEL), web applications (e.g., online software), and desktop software.

Table S2: Analysis and Classification of Reviewed Environmental Risk Assessment Frameworks.

Name of the framework and purpose	Intrinsic characteristics	Input	Output	Address data gaps[(a)]	Software tools[(b)]
Life Cycle Analysis [8-12]<br>*Application:* ENMs, ENM containing products	LCA refers to a class of approaches for assessing environmental impacts of ENMs, whereby hazard identification, exposure assessment and risk characterization may be analyzed throughout the ENMS life. Depending on the scope of the analysis, as shown in case studies, the approach can be modified to estimate environmental impacts (e.g., associated with production of ENMs or nano enabled products or environmental release of the ENMs related to consumer products).	Depending on the scope/outcome of the analysis: a) global warming/CO_2 emissions (detailed steps of manufacturing processes and their CO_2 emissions) [12,13]; b) potentially affected fraction of aquatic organisms per unit mass of CNTs (EC_{50} values, degradation rates, partition coefficients, releases to environmental compartments and bioaccumulation factors) [10].	Depending on the design/desired outcome (e.g., environmental impacts from ENM production or release): Quantitative global warming potential (CO_2 emissions related to the synthesis of the assessed ENM) or potential detrimental impacts on aquatic organisms as a consequence of the ENM release.	No	LCA general software (not specific for ENMs)
DUPONT's nanorisk [14]<br>*Application:* Processes	Combines a systematic collection and organization of information with a chemical process risk assessment (CPQRA) [15], when information is available. CPQRA** focuses on acute rather than chronic hazards. Risk = F (s,c,f), s= hypothetical scenario, c=estimated consequence(s), f=estimated frequency	Inputs include ENM properties (*e.g.*, name(s), form, chemical composition, surface coatings, molecular and crystal structure, physical form/shape, particle size, size distribution, surface-area, particle density, solubility (in water and biologically relevant fluids), dispersibility) and information about the industrial processes relevant to the ENM.	Depending on the available information, the results can be presented as lifecycle profiles that include information on physicochemical properties, ecotoxicity, and environmental fate to be used for risk management strategies; or include a quantitative risk analysis of the industrial processes related to the ENM.	No	No
EPA's Comprehensive Environmental Assessment CEA [16,17]<br>*Application*: ENMs	Collective "judgment process" designed to compile information and provide guidance to decision makers such as research planners and risk managers. The framework is presented as a roadmap to guide the user through the process of systematic data collection and identification of critical data gaps.	Product lifecycle, environmental fate and transport, exposure routes, in addition to dose related information (e.g., toxicokinetics: absorption, distribution, metabolism, and excretion), if available [18].	Summary of available information regarding a specific ENM evaluated by a group of experts indicating recommendations for research priorities and risk management.	No	Web application

Name of the framework and purpose	Intrinsic characteristics	Input	Output	Address data gaps[(a)]	Software tools[(b)]
Ranking initial environmental and human health risk: Nano HAZ framework [19] *Application:* ENMs	Ecological and human health risk assessment adapted to ENMs and application of benchmark dose (BMD) calculations based on published data.	Predicted nanomaterial environmental concentrations from published exposure studies (published modelled data [20]). Published ecotoxicological studies were used to develop provisional benchmark dose lower confidence limits (BMDLs) through the application of the US EPA Bench Mark Dose Software. A similar approach was conducted with animal studies to obtain BMDLs for human health risk calculations.	Classification/categories of the ENMs: Relative Risk Ranking groups. 0–2 (low environmental or health risk on a relative basis), 3–4 (concentrations that require monitoring and potential action), 5 + (environmental concentration above those provisional regulatory and toxicological limits as set in a published case study [19]).	Yes	No
Nanomaterial risk screening [21] *Application*: ENMs	The framework provides a template for the analyst in order to compare data for the target ENM with reference information for each category/level. Framework was developed via expert judgment.	ENM properties categorized as intrinsic (chemical composition, crystal structure, size (average), shape (aspect ratio), charge (zeta potential), specific surface area) and extrinsic properties (reactivity, solubility, hydrophobicity, agglomeration, and sorption tendency). Hazard indicators: ROS potential, movement through cells; Exposure indicators: persistence, and mobility.	Classification/categories of the ENMs: risk-rating groups (from lowest concern 1 to highest concern 5).	No	Spreadsheet
Human health and Ecological Risk Assessment as adapted from REACH [22] *Application:* ENMs	Follows the steps of a risk assessment for 90-day exposure studies and modeled environmental concentrations (from probabilistic material flow analysis (PMFA)).	Dose descriptors, overall assessment factors and estimated human indicative no-effect level (INELs) for workers of different ENM for chronic inhalation exposure based on experimental data and modeled environmental releases of ENMs.	Quantitative: Risk Quotient obtained by comparing predicted environmental concentrations with human no effect levels (PEC/INEC).	No	No
Risk quantification based on probabilistic flow modeling analysis [23] *Application:* ENMs	Risk assessment combines predicted environmental concentrations (via PMFA) with a species sensitivity distribution (*e.g.*, probability distribution of harmful effects as a function of concentration for a given ENM).	Probability distributions of ENMs environmental concentrations (obtained via PMFA) with the probability distribution of adverse effects developed from literature review (species sensitivity distributions SSD).	Quantitative: Risk Index calculated as the product of probability of critical environmental concentrations and the probability that organisms would potentially be negatively impacted at such concentrations.	Yes	No

**Name of the framework and purpose**	**Intrinsic characteristics**	**Input**	**Output**	**Address data gaps[(a)]**	**Software tools[(b)]**
FINE (Forecasting the Impacts of Nanomaterials in the Environment applied to nanoAg) [24] using Bayesian Networks *Application:* ENMs	Probability of risk calculations based on Bayes' principle; the network was designed via expert judgment.	Particle behavior under a set of aquatic and sediment environmental conditions (e.g., temperature, pH, fluid flow, organic matter, conductivity, time) in addition to NP properties (e.g., NP coating, zeta potential, fractal dimension, NP diameter, collision rate efficiency, homogeneous and heterogeneous NP attachment efficiencies, NP aggregation potential, biodegradation factors, deposition and dissolution) and the interaction surfaces; 2) Exposure related parameters (e.g., NP concentrations (including dissolved form) in water and sediment; 3) Hazard (Bioavailability potential, amount of bio-uptake, stage of development, mortality, growth/fitness, effects on microbial biomass in sediment and water, trace metal presence, reduction in decomposer community redundancy, reduction in denitrifier community redundancy, overall sediment community, overall water community, effect on decomposition***, methanogenesis, denitrification, primary production, carbon sequestration, trace gas emmissions, and eutrophication).	Quantitative: a modified version of the deterministic Risk Quotient shown as a probabilistic expression (probability measure scale of 0-1).	Yes	Software generic (not specific for ENMs)

** The guidelines described by the American Institute of Chemical Engineers AICHE and or the Health and Safety Executive of the UK. ***To analyze the effect of Nano Ag in the microbial community, the parameters included the effect on the microbial decomposition of organic matter, reduction in decomposer and denitrifier community redundancy, as well as the overall microbial community in the sediment [25].

Table S3: Analysis and Classification of Reviewed Occupational Risk Assessment Frameworks.

**Name of the framework and purpose**	**Intrinsic characteristics**	**Input**	**Output**	**Address data gaps[(a)]**	**Software tools[(b)]**
Risk based classification for occupational exposure control [26] *Application:* ENMs	The process follows a quantitative risk assessment (QRA) approach based on benchmark doses (BMD).	Particle size, shape, and density utilized in estimation of inhalation and lung region-specific deposition fraction; toxicity assays (multiple exposure or dose groups to describe dose–response relationship; estimated benchmark dose); biological significance of response (to evaluate severity and relevance to humans); body and lung weight; target lung region surface area and volume (to normalize dose from animals to humans).	Quantitative: Excess Risk - defined as the percent of excess risk for a specific health outcome.	Yes	No
Risk classification based on an Industry Insurance Protocol [27] *Application*: Processes	Comparison of scores assigned to characterize the industrial process of manufacturing ENMs with pre-established scores based on an insurance protocol.	Qualitative relative risk ranking that requires data on physicochemical properties and quantities of inventoried materials; relative risk assessment is also based on factors such as toxicity, flammability, and persistence in the environment. Additional required data include toxicity metrics (*e.g.*, LC_{50} and LD_{50}), and fate and transport realted information (*e.g.*, water solubility, log K_{ow}, flammability, and expected emissions).	Classification/categories: relative risk ranking - a comparison of an industrial chemical process vs. an ENM process.	Yes	No
Control Banding: CB Nanotool [28] *Application:* Processes	Classification based on the characteristics of the process and the hazard evidence for the target ENM.	ENM properties (surface chemistry, particle shape, particle diameter, solubility), toxicity evidence (carcinogenicity, reproductive toxicity, mutagenicity, dermal toxicity, toxicity of parent material (bulk material, non nano sized material considered similar to the ENM); estimated amount of ENM used for a given task, dustiness/mistiness, number of employees with similar exposure, frequency of operation, and duration of operation.	Classification/categories: Risk Level for occupational risk expressed as risk bands that indicate recommendations regarding the pursuit of risk management strategies needed for exposure control.	Yes	Spreadsheet (not publicly available)
Web-Based Tool for Risk Prioritization of Airborne Manufactured Nano Objects (Stoffenmanager Nano) [29] *Application:* Processes	Classification based on the characteristics of the process and the hazard evidence for the target ENM.	ENM properties (*e.g.*, particle shape, diameter, length, solubility, composition, bioavailability, reactivity); toxicity evidence (*e.g.*, carcinogenicity, reproductive toxicity, mutagenic); industrial process parameters (*e.g.*, duration and frequency of material handling, background concentration), characteristics of the ENM matrix (*e.g.*, dustiness of powder and ENM fraction in powder) and engineering controls (*e.g*, use of personal protective equipment).	Classification/categories: Priority bands that indicate the priority for risk management.	No	Web application

REFERENCES

1. Höck J., E.T., Furrer E., Gautschi. M, Hofmann H., Höhener K., Knauer K., Krug H., Limbach; L., G.P., Nowack B., Riediker M., Schirmer K., Schmid K., Som C., Stark W., Studer C., Ulrich A.,; von Götz N., W.A., Wengert S., Wick P., *Guidelines on the Precautionary Matrix for Synthetic Nanomaterials*. 2013, Federal Office of Public Health and Federal Office for the Environment: Berne.
2. Linkov, I.; Satterstrom, F.K.; Steevens, J.; Ferguson, E.; Pleus, R.C., *J. Nanopart. Res.*, 2007. **9**(4): p. 543-554.
3. Linkov, I.; Steevens, J.; Chappell, M.; Tervonen, T.; Figueira, J.R.; Merad, M., *Classifying Nanomaterial Risks Using Multi-Criteria Decision Analysis*, in *Nanomaterials: Risks and Benefits*, Linkov, I.Steevens, J., Editors. 2009, Springer Netherlands. p. 179-191.
4. Tervonen, T.; Linkov, I.; Figueira, J.R.; Steevens, J.; Chappell, M.; Merad, M., *J. Nanopart. Res.*, 2009. **11**(4): p. 757-766.
5. Hansen, S.F.; Jensen, K.A.; Baun, A., *J. Nanopart. Res.*, 2014. **16**(1): p. 1-25.
6. Arts, J.H.E.; Hadi, M.; Irfan, M.-A.; Keene, A.M.; Kreiling, R.; Lyon, D.; Maier, M.; Michel, K.; Petry, T.; Sauer, U.G.; Warheit, D.; Wiench, K.; Wohlleben, W.; Landsiedel, R., *Regul. Toxicol. Pharmacol.*, 2015. **71**(2, Supplement): p. S1-S27.
7. Sass, J.; Heine, L.; Hwang, N., *Environ. Health*, 2016. **15**(1): p. 105.
8. Som, C.; Berges, M.; Chaudhry, Q.; Dusinska, M.; Fernandes, T.F.; Olsen, S.I.; Nowack, B., *Toxicology*, 2010. **269**(2-3): p. 160-169.
9. Theis, T.L.; Bakshi, B.R.; Durham, D.; Fthenakis, V.M.; Gutowski, T.G.; Isaacs, J.A.; Seager, T.; Wiesner, M.R., *Phys. Status Solidi-Rapid Res. Lett.*, 2011. **5**(9): p. 312-317.
10. Eckelman, M.J.; Mauter, M.S.; Isaacs, J.A.; Elimelech, M., *Environ. Sci. Technol.*, 2012. **46**(5): p. 2902-2910.
11. Gavankar, S.; Suh, S.; Keller, A.F., *Int. J. Life Cycle Assess.*, 2012. **17**(3): p. 295-303.
12. Hischier, R.; Walser, T., *Sci. Total Environ.*, 2012. **425**.
13. Walser, T.; Demou, E.; Lang, D.J.; Hellweg, S., *Environ. Sci. Technol.*, 2011. **45**(10): p. 4570-4578.
14. DUPONT. *Nanorisk Framework*. 2007; http://www.nanoriskframework.org/.
15. AIChE, *Guidelines for Chemical Process Quantitative Risk Analysis (2nd Edition)*. 2000, Center for Chemical Process Safety/AIChE.
16. Powers, C.M.; Dana, G.; Gillespie, P.; Gwinn, M.R.; Hendren, C.O.; Long, T.C.; Wang, A.; Davis, J.M., *Environ. Sci. Technol.*, 2012. **46**(17): p. 9202-9208.
17. Powers, C.M.; Grieger, K.D.; Hendren, C.O.; Meacham, C.A.; Gurevich, G.; Lassiter, M.G.; Money, E.S.; Lloyd, J.M.; Beaulieu, S.M., *Sci. Total Environ.*, 2014. **470**: p. 660-668.
18. EPA, U., *Comprehensive Environmental Assessment Applied to Multiwalled Carbon Nanotube Flame-Retardant Coatings in Upholstery Textiles: A Case Study Presenting Priority Research Gaps for Future Risk Assessments (Final Report)*. 2013.
19. O'Brien, N.; Cummins, E., *Journal of Environmental Science and Health Part a-Toxic/Hazardous Substances & Environmental Engineering*, 2010. **45**(8): p. 992-1007.
20. Gottschalk, F.; Scholz, R.W.; Nowack, B., *Environ. Modell. Software*, 2010. **25**(3).
21. Beaudrie, C.E.H.; Kandlikar, M.; Gregory, R.; Long, G.; Wilson, T., *Environment Systems & Decisions*, 2015. **35**(1, Sp. Iss. SI): p. 88-109.
22. Aschberger, K.; Micheletti, C.; Sokull-Kluttgen, B.; Christensen, F.M., *Environ. Int.*, 2011. **37**(6): p. 1143-1156.

23. Gottschalk, F.; Kost, E.; Nowack, B., *Environ. Toxicol. Chem.*, 2013. **32**(6): p. 1278-1287.
24. Money, E.S.; Reckhow, K.H.; Wiesner, M.R., *Sci. Total Environ.*, 2012. **426**: p. 436-445.
25. Goksøyr, J., *Decomposition, Microbiology, and Ecosystem Analysis*, in *Fennoscandian Tundra Ecosystems: Part 1 Plants and Microorganisms*, Wielgolaski, F.E., Editor. 1975, Springer Berlin Heidelberg: Berlin, Heidelberg. p. 230-238.
26. Kuempel, E.D.; Castranova, V.; Geraci, C.L.; Schulte, P.A., *J. Nanopart. Res.*, 2012. **14**(9).
27. Robichaud, C.O.; Tanzil, D.; Weilenmann, U.; Wiesner, M.R., *Environ. Sci. Technol.*, 2005. **39**(22).
28. Paik, S.Y.; Zalk, D.M.; Swuste, P., *Ann. Occup. Hyg.*, 2008. **52**(6): p. 419-428.
29. Van Duuren-Stuurman, B.; Vink, S.R.; Verbist, K.J.M.; Heussen, H.G.A.; Brouwer, D.H.; Kroese, D.E.D.; Van Niftrik, M.F.J.; Tielemans, E.; Fransman, W., *Ann. Occup. Hyg.*, 2012. **56**(5).
